# HIV status disclosure by Nigerian men who have sex with men and transgender women living with HIV: a cross-sectional analysis at enrollment into an observational cohort

**DOI:** 10.1186/s12889-020-09315-y

**Published:** 2020-08-26

**Authors:** Abdulwasiu B. Tiamiyu, John Lawlor, Fengming Hu, Afoke Kokogho, Manhattan E. Charurat, Charles Ekeh, Merlin L. Robb, Sylvia Adebajo, George Eluwa, Julie A. Ake, Stefan D. Baral, Rebecca G. Nowak, Trevor A. Crowell, for the TRUST/RV368 Study Group, Sylvia Adebajo, Stefan Baral, Trevor Crowell, Charlotte Gaydos, Afoke Kokogho, Jennifer Malia, Olumide Makanjuola, Nelson Michael, Nicaise Ndembi, Rebecca Nowak, Oluwasolape Olawore, Zahra Parker, Sheila Peel, Habib Ramadhani, Merlin Robb, Cristina Rodriguez-Hart, Eric Sanders-Buell, Elizabeth Shoyemi, Sodsai Tovanabutra, Sandhya Vasan

**Affiliations:** 1 HJF Medical Research International, Abuja, Nigeria; 2 U.S. Military HIV Research Program, Walter Reed Army Institute of Research, Silver Spring, MD USA; 3 Henry M. Jackson Foundation for the Advancement of Military Medicine, Bethesda, MD USA; 4 Institute of Human Virology, University of Maryland School of Medicine, Baltimore, MD USA; 5 Population Council Nigeria, Abuja, Nigeria; 6 Maryland Global Initiatives Corporation, University of Maryland, Baltimore, MD USA; 7 Johns Hopkins Bloomberg School of Public Health, Baltimore, MD USA

**Keywords:** HIV, Disclosure, Sexual and gender minorities, Nigeria, Social stigma, HIV management outcomes

## Abstract

**Background:**

Men who have sex with men (MSM) and transgender women (TGW) are disproportionately impacted by HIV and may face barriers to HIV status disclosure with negative ramifications for HIV prevention and care. We evaluated HIV status disclosure to sexual partners, HIV treatment outcomes, and stigma patterns of MSM and TGW in Abuja and Lagos, Nigeria.

**Methods:**

Previously-diagnosed MSM and TGW living with HIV who enrolled in the TRUST/RV368 cohort from March 2013 to August 2018 were asked, “Have you told your (male/female) sexual partners (MSP/FSP) that you are living with HIV?” In separate analyses, robust Poisson regression models were used to estimate risk ratios (RRs) and 95% confidence intervals (95% CIs) for characteristics associated with HIV status disclosure to MSP and FSP. Self-reported stigma indicators were compared between groups.

**Results:**

Of 493 participants living with HIV, 153 (31.0%) had disclosed their HIV status to some or all MSP since being diagnosed. Among 222 with FSP, 34 (15.3%) had disclosed to some or all FSP. Factors independently associated with disclosure to MSP included living in Lagos (RR 1.58 [95% CI 1.14–2.20]) and having viral load < 50 copies/mL (RR 1.67 [95% CI 1.24–2.25]). Disclosure to FSP was more common among participants who were working in entertainment industries (RR 6.25 [95% CI 1.06–36.84]) or as drivers/laborers (RR 6.66 [95% CI 1.10–40.36], as compared to unemployed) and also among those married/cohabiting (RR 3.95 [95% CI 1.97–7.91], as compared to single) and prescribed ART (RR 2.27 [95% CI 1.07–4.83]). No differences in self-reported stigma indicators were observed by disclosure status to MSP but disclosure to FSP was associated with a lower likelihood of ever having been assaulted (26.5% versus 45.2%, *p* = 0.042).

**Conclusions:**

HIV status disclosure to sexual partners was uncommon among Nigerian MSM and TGW living with HIV but was associated with improved HIV care outcomes. Disclosure was not associated with substantially increased experiences of stigma. Strategies to encourage HIV status disclosure may improve HIV management outcomes in these highly-marginalized populations with a high burden of HIV infection.

## Background

Sub-Saharan Africa is home to about 25.7 million people living with HIV (PLWH) and over two-thirds of the global burden of HIV incidence is in this region [1]. Nigeria is the most populous African country and has the second largest population of PLWH, accounting for 10% of new infections and 14% of AIDS-related deaths worldwide every year [2, 3]. HIV disproportionately impacts men who have sex with men (MSM) and transgender women (TGW) as compared to other reproductive aged adults [1]. Nigerian national estimates suggest that HIV prevalence among MSM is about 23%, which is 4–10 times higher than the general population [4, 5]. We have previously reported HIV prevalence of 44–66% among MSM and TGW receiving care at community-based health centers in Abuja and Lagos, Nigeria [6].

The disproportionate impact of HIV can be partly explained by stigmatization, discrimination, and homophobia that impede engagement of Nigerian MSM and TGW in HIV prevention and care services [7]. These factors perpetuate the HIV epidemic and drive some individuals to maintain both public heterosexual relationships and private same-sex ones [8]. Nigerian MSM and TGW rarely disclose their same-sex sexual practices to healthcare providers and family members, creating barriers to receipt of appropriate healthcare services such as HIV testing and anatomically appropriate screening for other sexually transmitted infections [9]. Stigma that is deep-rooted within Nigerian culture was codified into law in 2014, when Nigeria passed the Same Sex Marriage Prohibition Act, which prohibits marriage or civil union by persons of the same sex, solemnization of same sex marriage in places of worship, direct or indirect public displays of affection by same-sex couples and registration of homosexual clubs and societies [10–13]. The passage of this Act was supported by leaders of both major religions in Nigeria —Christianity and Islam [14]. Studies from other African countries have linked negative religious perspectives of same-sex sexual practices to increased stress and stigmatization [15, 16]. MSM and TGW who are living with HIV are subject to compounded and intersectional stigma due to both same-sex sexual practices and HIV [17–20].

Disclosure of HIV status may expose PLWH to societal rejection, stigma, persecution, and discrimination [17]. Among Nigerian cisgender women, risk of abandonment or separation and feelings of shame, worry and fear have been identified as barriers to HIV status disclosure in one prior study [21]. The paradox is that disclosure of HIV status offers potential individual and public health benefits. Disclosure can enhance adherence to antiretroviral therapy (ART), create social support, lessen anxiety, enhance partners’ HIV testing, facilitate engagement in care, and reduce condomless sex [22–26]. Disclosure to sexual partners enables informed discussion and decision-making surrounding the use of HIV prevention technologies such as condoms, pre-exposure prophylaxis, and treatment as prevention [27]. In a previous study of predominantly heterosexual Nigerian men and women living with HIV, about three-quarters reported that their sexual partners responded with support, understanding, or kindness upon HIV status disclosure [28]. Studies in other populations have found HIV status disclosure to be facilitated by factors such as ethical obligations or feelings of guilt, opportunities for romantic relationships, open bi-directional communications between sexual partners, support for disclosure provided by healthcare workers, and de-stigmatizing community education programs [29–32].

Understanding HIV status disclosure practices among Nigerian MSM and TGW could inform interventions to optimize delivery of HIV prevention and treatment services to this marginalized population with a high burden of HIV. Among Nigerian MSM and TGW living with HIV, we characterized HIV status disclosure patterns to sexual partners and evaluated downstream effects on HIV management outcomes and stigma indicators. We hypothesized that HIV status disclosure would be associated with both improved HIV management outcomes and potentially increased stigma.

## Methods

### Study population

The TRUST/RV368 cohort is a prospective observational study of MSM and TGW engaged in community-based HIV prevention and treatment programs in Abuja and Lagos, Nigeria. Recruitment utilized respondent-driven sampling (RDS), in which 12 “seed” participants were identified through local non-governmental organizations and key opinion leaders to represent diverse demographic and socioeconomic characteristics. Each “seed” referred up to three potential participants from their social networks in the MSM and TGW communities [33, 34]. Potential participants followed instructions on their referral coupons to present to the site for individualized study briefings prior to enrollment. Each enrolled participant could similarly refer up to three additional participants. To be enrolled, prospective participants had to present a valid RDS recruitment coupon indicating referral from a current study participant. Inclusion criteria for enrollment also included male sex at birth; age of at least 16 years in Abuja or 18 years in Lagos (reflecting differences in local IRB guidance); and self-reported insertive or receptive anal sex with a male partner in the last year. Compensation was provided for both participation in study visits (Naira 2000–3400 [approximately US$6–11] depending on visit) and for successful referrals (Naira 1500 [approximately US$5]).

These analyses included MSM and TGW who enrolled from March 2013 to August 2018, were previously known to be living with HIV, and answered the survey question about HIV status disclosure to male sexual partners.

### Data collection

Data for these cross-sectional analyses were collected at enrollment into the TRUST/RV368 cohort with evaluations spread across two visits approximately 2 weeks apart. Participant demographics (age, gender identity, sexual orientation, education level, occupation, and marital status), perceptions and experiences of stigma, and sexual behaviors were evaluated using questionnaires developed specifically for this this study (Supplementary Files 1–2) that were administered during a face-to-face interview with a specially-trained staff member lasting approximately 1–2 h at each visit [19]. Participants were asked up to two questions about HIV status disclosure(s) since becoming aware that they were living with HIV. First, all participants were asked, “Have you told your male sexual partners (MSP) that you are living with HIV?” Participants answered “yes, all male sexual partners”, “some but not all male sexual partners”, or “no, none”. If a participant reported female sexual partners (FSP), the participant was also asked, “Have you told your female sexual partners that you are living with HIV?” and provided similar answer choices. For these analyses, disclosure was dichotomized as disclosure to some/all partners or none. Perceived and experienced stigma were assessed using a variety of self-reported indicators including lifetime experience of fear of accessing healthcare services, avoidance of healthcare, denial of healthcare, verbal harassment, assault, and forced sex due to same-sex sexual practices.

HIV status was confirmed using a parallel algorithm of rapid tests with Determine® (Alere, Waltham, MA, USA) and Uni-gold® (Trinity Biotech, Co-Wicklow, Ireland) kits plus, if needed, a tie-breaker STAT-PAK (Chembio, NY, USA) kit. HIV viral load was quantified using the COBAS TaqMan HIV-1 Test (Roche Molecular Diagnostics, Pleasanton, CA). CD4 count was estimated using the Partec CyFlow Counter (Sysmex, Lincolnshire, IL). A study clinician reviewed existing medical records and took a detailed medical history from each participant, including a chronicle of any prior ART use.

### Statistical analyses

All participants included in these analyses reported MSP and were stratified based on whether or not they reported disclosing their HIV status to MSP. Among participants who reported FSP, stratification was also performed based on self-reported disclosure of HIV status to FSP. Variables were pre-selected based on review of prior literature about disclosure patterns. Categorical variables of interest were compared between these groups using Pearson’s Chi-square test or, in cases with small cell sizes, the exact Chi-square test. In separate analyses by sexual partner type, unadjusted and adjusted robust Poisson regression models were used to estimate relative risks (RRs) and 95% confidence intervals (CIs) for pre-specified factors potentially associated with HIV status disclosure [35]. All analyses were performed using SAS version 9.04 (SAS Institute Inc., Cary, NC, USA).

## Results

### Study population

A total of 2737 participants were enrolled. Of these, 984 were living with HIV, 529 knew their HIV status prior to enrollment, and 493 answered the question about disclosure to MSP. Of these, 222 also reported FSP, all of whom answered the question about disclosure to FSP. The median age of participants in these analyses was 25 (interquartile range [IQR] 22–29) years. Only 153 of 493 (31.0%) disclosed their HIV status to some or all MSP and 34 of 222 who reported FSP (15.3%) disclosed to some or all FSP (Table 1).

**Table 1:** Demographic characteristics of previously-diagnosed Nigerian men who have sex with men and transgender women living with HIV

Characteristics	**Disclosed to Male Sexual Partners**		***P***	**Disclosed to Female Sexual Partners**		P*
	None (*n* = 340)	Some/All (*n* = 153)		None (*n* = 188)	Some/All (*n* = 34)	
**Age**						
≤ 21 years	59 (70.2)	25 (29.8)	0.700	20 (87.0)	3 (13.0)	**< 0.001**
22–30 years	229 (69.6)	100 (30.4)		135 (90.6)	14 (9.4)	
> 30 years	52 (65.0)	28 (35.0)		33 (66.0)	17 (34.0)	
**Gender Identity**						
Cisgender Man	249 (67.3)	121 (32.7)	0.310	155 (85.6)	26 (14.4)	0.724
Transgender Woman	49 (76.6)	15 (23.4)		14 (77.8)	4 (22.2)	
Other/Unknown	42 (71.2)	17 (28.8)		19 (82.6)	4 (17.4)	
**Sexual Orientation**						
Gay/Homosexual	110 (67.1)	54 (32.9)	0.794*	10 (76.9)	3 (23.1)	0.743
Bisexual	228 (69.9)	98 (30.1)		177 (85.1)	31 (14.9)	
Other/Unknown	2 (66.7)	1 (33.3)		1 (100)	0 (0.0)	
**Education Level**						
Junior Secondary or Less	16 (55.2)	13 (44.8)	0.083*	11 (78.6)	3 (21.4)	0.890
Senior Secondary	187 (73.6)	67 (26.4)		95 (84.8)	17 (15.2)	
Higher than Senior Secondary	134 (65.4)	71 (34.6)		81 (85.3)	14 (14.7)	
Unknown	3 (60.0)	2 (40.0)		1 (100.0)	0 (0.0)	
**Occupation**						
Unemployed	50 (69.4)	22 (30.6)	0.587	24 (96.0)	1 (4.0)	0.122
Student	48 (75.0)	16 (25.0)		26 (96.3)	1 (3.7)	
Professional/Self-Employed	40 (64.5)	22 (35.5)		25 (86.2)	4 (13.8)	
Entertainment/Hospitality	32 (78.0)	9 (22.0)		13 (72.2)	5 (27.8)	
Driver/Laborer	6 (66.7)	3 (33.3)		5 (83.3)	1 (16.7)	
Other/Unknown	164 (66.9)	81 (33.1)		95 (81.2)	22 (18.8)	
**Marital Status**						
Single/Never Married	306 (70.7)	127 (29.3)	0.062	169 (90.4)	18 (9.6)	**< 0.001**
Married/Cohabiting	23 (60.5)	15 (39.5)		12 (46.2)	14 (53.8)	
Divorced/Widowed/Other	11 (50.0)	11 (50.0)		7 (77.8)	2 (22.2)	
**Site**						
Abuja	210 (70.9)	86 (29.1)	0.244	127 (82.5)	27 (17.5)	0.225
Lagos	130 (66.0)	67 (34.0)		61 (89.7)	7 (10.3)	

All data are presented as n (%). Comparisons were made between groups using Pearson’s Chi-square test or, if indicated by an asterisk (*), exact Chi-square test. Statistically significant *p*-values (*p* ≤ 0.05) are in **bold**

### Disclosure patterns

Among participants who reported FSP, those who disclosed their HIV status to MSP were more likely to also disclose to FSP as compared to those who did not disclose to MSP (25.4% versus 11.7%, *p* = 0.012). Stated another way, participants who disclosed their HIV status to FSP were more likely to also disclose to MSP as compared to those who did not disclose to FSP (44.1% versus 23.4%, *p* = 0.012). A total of 15 participants disclosed to both MSP and FSP.

Participants who disclosed their HIV status to MSP were more likely to disclose to healthcare providers (88.9% versus 76.2%, *p* = 0.001). However, the proportion who disclosed to healthcare providers did not differ significantly between participants who did and did not disclose to FSP (82.4% versus 77.1%, *p* = 0.499).

### HIV care cascade

**Fig. 1:**
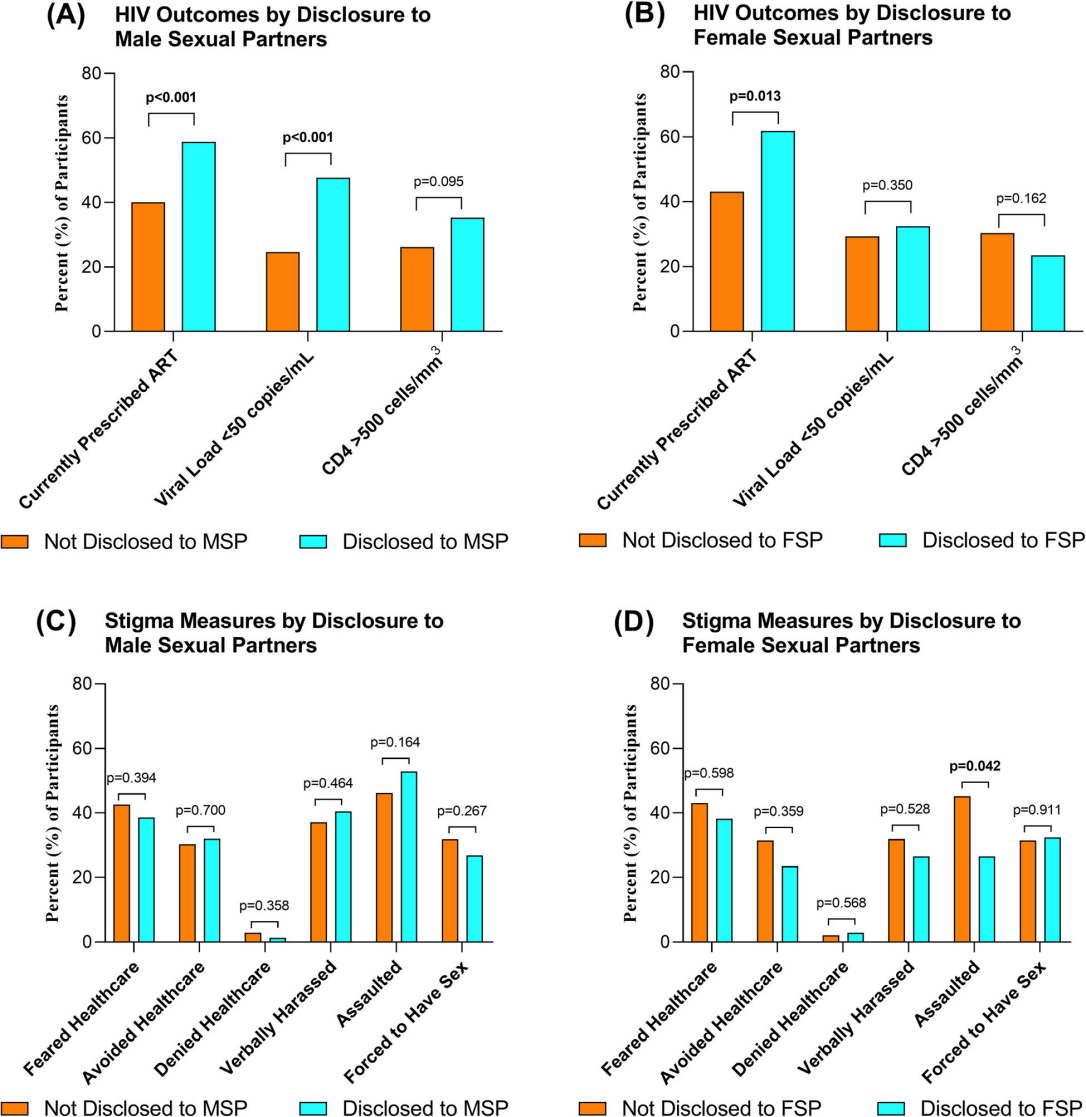
Associations of HIV Status Disclosure with HIV Care Outcomes and Self-Reported Stigma among Nigerian Men who have Sex with Men and Transgender Women. Medical records were reviewed at study enrollment to ascertain ART prescription status. HIV viral load and CD4 count were enumerated for all enrolled participants living with HIV. Participants were asked whether or not they had ever experienced a variety of specific indicators of anticipated and enacted stigma. The percentage of participants demonstrating each key HIV care outcome is presented with stratification by disclosure of HIV status to male sexual partners (Panel **a**) and to female sexual partners (Panel **b**). The percentage of participants who reported each stigma indicator is presented with stratification by disclosure of HIV status to male sexual partners (Panel **c**) and to female sexual partners (Panel **d**). Statistically significant *p*-values (*p* ≤ 0.05) for comparisons by disclosure status are in **bold**

Compared to those who had not disclosed their HIV status to MSP, participants who had disclosed were more likely to be prescribed ART (58.8% versus 40.0%, *p* < 0.001) and to have viral load < 50 copies/mL (47.7% versus 24.7%, *p* < 0.001; Fig. 1a). There was no difference in the proportion of participants with CD4 > 500 cells/mm^3^ based on having disclosed or not to MSP (35.4% versus 26.2%, *p* = 0.095), though median CD4 was higher in the group that had disclosed (438 [IQR 327–572] versus 384 [IQR 270–537] cells/mm^3^, *p* = 0.032). Participants who had disclosed their status to FSP were more likely to be prescribed ART (61.8% versus 43.1%, *p* = 0.013; Fig. 1b). There were no statistically significant differences between participants who had and had not disclosed their HIV status to FSP in terms of viral suppression (32.4% versus 29.3%, *p* = 0.350), CD4 > 500 cells/mm^3^ (23.5% versus 30.3%, *p* = 0.162), or median CD4 (337 [IQR 172–651] versus 409 [IQR 267–558] cells/mm^3^, *p* = 0.219).

### Stigma

The prevalence of self-reported stigma indicators was similar between participants who had disclosed their HIV status to MSP and those who had not (Fig. 1c). Participants who disclosed their HIV status to FSP were less likely to report assault than were participants who had not disclosed to FSP (26.5% versus 45.2%, *p* = 0.042), but other indicators of stigma did not vary by disclosure status to FSP (Fig. 1d).

### Factors associated with HIV status disclosure

After adjusting for other factors, HIV status disclosure to MSP was more likely among participants at the Lagos site (RR 1.58 [95% CI 1.14–2.20, *P* = 0.006]) or who were virally suppressed (RR 1.67 [95% CI 1.24–2.25, *P* < 0.001]) and less likely with increasing education levels (RR 0.63 [95% CI 0.41–0.95, *P* = 0.029]) (Table 2). HIV status disclosure to FSP was more likely among participants that were married or living with a woman (RR 3.95 [95% CI 1.97–7.91, *P* < 0.001]), were working in entertainment industries (RR 6.25 [95% CI 1.06–36.84, *P* = 0.043]) or as a driver/laborer (RR 6.66 [95% CI 1.10–40.36, *P* = 0.039]), were prescribed ART (RR 2.27 [95% CI 1.07–4.83, *P* = 0.033]), and had disclosed to MSP (RR 1.93 [95% CI 1.02–3.67, *P* = 0.043]).

**Table 2:** Factors associated with HIV status disclosure to male and female sexual partners of Nigerian men who have Sex with Men and Transgender Women

Characteristics	**Disclosure to Some/All Male Sexual Partners** Relative Risk (95% Confidence Interval)				**Disclosure to Some/All Female Sexual Partners** Relative Risk (95% Confidence Interval)			
	Unadjusted	p	Adjusted	p	Unadjusted	p	Adjusted	p
**Age**								
≤ 21 years	Reference		Reference		Reference		Reference	
22–30 years	1.02 (0.71–1.47)	0.910	0.88 (0.61–1.28)	0.512	0.72 (0.22–2.31)	0.582	0.45 (0.13–1.53)	0.201
> 30 years	1.18 (0.75–1.83)	0.474	0.74 (0.46–1.19)	0.213	2.61 (0.85–8.02)	0.095	0.87 (0.25–3.10)	0.835
**Gender Identity**								
Man	Reference		Reference		Reference		Reference	
Woman	0.72 (0.45–1.14)	0.161	0.67 (0.43–1.05)	0.078	1.55 (0.61–3.94)	0.360	1.58 (0.62–4.04)	0.336
Other/Unknown	0.88 (0.57–1.35)	0.561	0.89 (0.58–1.36)	0.594	1.21 (0.46–3.16)	0.696	1.59 (0.60–4.25)	0.351
**Sexual Orientation**								
Gay/Homosexual^a^	Reference		Reference		Reference		Reference	
Bisexual	0.91 (0.70–1.20)	0.512	1.01 (0.76–1.34)	0.952	0.70 (0.24–2.00)	0.500	0.62 (0.17–2.28)	0.474
**Education Level**								
Junior Secondary or less^a^	Reference		Reference		Reference		Reference	
Senior Secondary	**0.60 (0.39–0.92)**	**0.019**	**0.47 (0.30–0.73)**	**< 0.001**	0.76 (0.25–2.29)	0.624	1.24 (0.51–3.03)	0.634
Higher than Senior Secondary	0.79 (0.51–1.20)	0.262	**0.63 (0.41–0.95)**	**0.029**	0.74 (0.24–2.26)	0.594	1.40 (0.55–3.56)	0.476
**Occupation**								
Unemployed	Reference		Reference		Reference		Reference	
Student	0.82 (0.47–1.42)	0.474	0.78 (0.46–1.33)	0.369	0.93 (0.06–14.03)	0.956	1.62 (0.15–17.42)	0.689
Professional/Self-Employed	1.16 (0.72–1.88)	0.545	0.97 (0.59–1.59)	0.902	3.45 (0.41–28.87)	0.254	2.84 (0.48–16.70)	0.247
Entertainment/Hospitality	0.72 (0.37–1.41)	0.336	0.87 (0.44–1.72)	0.685	6.94 (0.89–54.47)	0.065	**6.25 (1.06–36.84)**	**0.043**
Driver/Laborer	1.09 (0.41–2.93)	0.863	0.92 (0.41–2.07)	0.834	4.17 (0.30–57.50)	0.287	**6.66 (1.10–40.36)**	**0.039**
Other/Unknown/Missing	1.08 (0.73–1.60)	0.693	1.24 (0.80–1.91)	0.334	4.70 (0.66–33.27)	0.121	4.06 (0.84–19.74)	0.082
**Marital Status**								
Single/Never Married	Reference		Reference		Reference		Reference	
Married/Cohabiting	1.35 (0.88–2.05)	0.166	1.16 (0.71–1.90)	0.545	**5.59 (3.18–9.84)**	**< 0.001**	**3.95 (1.97–7.91)**	**< 0.001**
Divorced/Widowed/Other	**1.70 (1.09–2.65)**	**0.018**	1.29 (0.86–1.94)	0.210	2.31 (0.63–8.46)^3^	0.207	1.26 (0.31–5.08)^3^	0.748
**Site**								
Abuja	Reference		Reference		Reference		Reference	
Lagos	1.17 (0.90–1.52)	0.242	**1.58 (1.14–2.20)**	**0.006**	0.59 (0.27–1.28)	0.181	1.03 (0.33–3.25)	0.960
**CD4 Count**								
< 500 cells/mm^3^	Reference		Reference		Reference		Reference	
≥ 500 cells/mm^3^	1.31 (0.99–1.72)	0.055	1.10 (0.83–1.45)	0.496	0.86 (0.40–1.85)	0.691	0.85 (0.42–1.70)	0.639
Unknown/Missing	0.82 (0.47–1.46)	0.505	1.31 (0.71–2.41)	0.381	1.95 (0.92–4.13)	0.084	2.53 (0.99–6.44)	0.051
**Viral Load**								
> =50 copies/mL	Reference		Reference		Reference		Reference	
< 50 copies/mL	**1.87 (1.44–2.43)**	**< 0.001**	**1.67 (1.24–2.25)**	**< 0.001**	1.28 (0.64–2.58)	0.482	0.61 (0.26–1.41)	0.247
Unknown/Missing	0.67 (0.33–1.36)	0.268	0.57 (0.23–1.39)	0.218	1.85 (0.81–4.23)	0.145	0.77 (0.23–2.53)	0.667
**Currently Prescribed ART**								
No	Reference		Reference		Reference		Reference	
Yes	**1.67 (1.27–2.19)**	**< 0.001**	1.32 (0.93–1.86)	0.117	1.56 (0.99–2.44)	0.053	**2.27 (1.07–4.83)**	**0.033**
Unknown/Missing	0.76 (0.21–2.72)	0.677	1.34 (0.29–6.13)	0.706	0.58 (0.09–3.77)	0.572	1.82 (0.48–6.87)	0.377
**Disclosed to Healthcare Provider**								
No	Reference		Reference		Reference		Reference	
Yes, any HCP	**1.98 (1.26–3.12)**	**0.003**	1.51 (0.93–2.46)	0.097	**3.06 (1.30–7.23)**	**0.011**	1.09 (0.42–2.82)	0.857
**Disclosed to Male Sexual Partners**								
No	Not Applicable		Not Applicable		Reference		Reference	
Some or all MSP					**2.18 (1.19–4.01)**	**0.012**	**1.93 (1.02–3.67)**	**0.043**
**Disclosed to Female Sexual Partners**								
No	Reference		Reference		Not Applicable		Not Applicable	
Some or all FSP	**1.54 (1.04–2.26)**	**0.030**	1.45 (0.97–2.18)	0.069				
Not Applicable (No FSP)	1.14 (0.81–1.62)	0.447	1.21 (0.82–1.79)	0.338				

*Abbreviations*: *ART* antiretroviral therapy, *HCP* healthcare provider, *FSP* female sexual partner, *MSP* male sexual partner

^a^To enable convergence of the models, “other/unknown” was combined into the reference group for sexual orientation and education level

Modified Poisson regression models were used to calculate relative risk and 95% confidence intervals for factors associated with HIV status disclosure. All variables in unadjusted models were included in the adjusted model for disclosure to each sex. All 493 participants included in these analyses reported male sexual partners in the preceding 12 months and contributed data to the models for disclosure to male sexual partners. Only the 222 participants who reported female sexual partners in the preceding 12 months contributed data to the models for disclosure to female sexual partners. Statistically significant associations (*p* ≤ 0.05) are shown in **bold**

## Discussion

We found that HIV status disclosure was relatively uncommon to sexual partners of Nigerian MSM and TGW living with HIV. Our finding that about one-third disclosed to MSP and one-sixth to FSP is similar to the 33% of MSM who disclosed their HIV status to sexual partners in one internet-based study in Asia [36] but much lower than the approximately 70% of MSM living with HIV in the United States who have disclosed to their partners [37, 38]. HIV-related stigma is one potential barrier to HIV status disclosure, which has been extensively documented among Nigerian MSM and TGW and can intersect with stigmas related to same-sex sexual practices [17, 21, 39]. These intersecting stigmas likely contributed to the lower prevalence of HIV status disclosure in our study as compared to the US, where stigma related to both HIV and same-sex sexual practices is less severe [40]. Notably, Nigeria passed an antidiscrimination bill into law in early 2015 that aimed to protect the rights and dignity of PLWH [41], but did so months after passing a law that criminalized same-sex sexual practices and relationships [42].

In our study, HIV status disclosure to sexual partners was associated with improved ART uptake, greater likelihood of viral suppression, and increased CD4 count. This finding is consistent with previous data among PLWH that suggested that disclosure improved access to HIV care and treatment [22–24, 27]. In addition, disclosure has been associated with reduced frequency of condomless sex and other behaviors associated with HIV transmission and acquisition [23, 24]. Facilitating HIV status disclosure could be a useful component of comprehensive care models to improve individual health of PLWH and decrease HIV transmission risk.

There is concern that potential beneficial effects of disclosure could be offset by adverse consequences such as societal rejection, persecution, discrimination, abandonment, separation and feelings of shame, worry or fear [17, 21, 39]. MSM and TGW living with HIV may face additional stigma due to same-sex sexual practices [9, 17–20]. However, we found few differences in self-reported stigma based on disclosure status. In fact, disclosure to FSP was associated with decreased risk of assault, a desirable indicator of decreased stigma. These findings suggest that safe spaces for disclosure of HIV status exist within some individual sexual partnerships of PLWH attending trusted community healthcare centers in Lagos and Abuja, Nigeria. Continued education and counseling to facilitate discussion and disclosure of HIV status should be provided to PLWH and those in serodiscordant partnerships.

These analyses were strengthened by an RDS recruitment strategy that enabled enrollment and characterization of hard-to-reach populations and a standardized questionnaire that enabled thorough description of demographic characteristics, disclosure patterns, and stigma indicators. However, these analyses may have been susceptible to response and reporting biases from self-reporting of sensitive and potentially stigmatizing information to study interviewers. Stigma indicators were constructed around perceived and experienced stigma due to same-sex sexual practices, which may be different from any stigma experienced due to HIV status. Furthermore, these analyses used pooled data from populations independently recruited in two cities using RDS to evaluate internal relationships between HIV status disclosure and other variables of interest; they did not account for any sampling bias introduced through the RDS recruitment methodology. Recruitment spanned a five-year period and longitudinal changes in HIV status disclosure practices were not evaluated in these cross-sectional analyses but could have been influenced by factors such as passage of the Same-Sex Marriage Prohibition Act in 2014. Findings from participants recruited at community health centers catering to sexual and gender minorities in Nigerian urban centers may not be generalizable to populations unwilling or unable to access care at such dedicated facilities.

## Conclusions

This study demonstrated complex relationships between of HIV status disclosure, stigma, and HIV care outcomes among Nigerian MSM and TGW attending trusted community healthcare centers in Lagos and Abuja. Cultural and behavioral implications of HIV status disclosure must be carefully considered when designing interventional strategies to engage MSM and TGW in HIV prevention and treatment. Interventions that encourage HIV status disclosure should be accompanied by community education about HIV, creation of safe spaces for PLWH to access care, and promotion of policies to encourage acceptance of PLWH in their communities. Similar sensitization to the needs of gender and sexual minorities must also be conducted. Improved HIV status disclosure practices are necessary to improve HIV care uptake and decrease HIV transmission among key populations.

## Supplementary information

**Additional file 1: Supplementary File 1.** TRUST/RV368 Questionnaire (Visit 0). Participant demographics, perceptions and experiences of stigma, and sexual behaviors were evaluated using questionnaires developed specifically for this study that were administered by a trained interviewer over the first two study visits, approximately two weeks apart. The first set of questions (Visit 0) covered demographic and socioeconomic information; human rights and exposure to violations; disclosure of same-sex sexual practices; risk behavior with sexual partners; depression; and knowledge, attitudes, and behavior.

**Additional file 2: Supplementary File 2.** TRUST/RV368 Questionnaire (Visit 1). Participant demographics, perceptions and experiences of stigma, and sexual behaviors were evaluated using questionnaires developed specifically for this study that were administered by a trained interviewer over the first two study visits, approximately two weeks apart. The second set of questions (Visit 1) covered sexual network risks; composition and influence of social networks; condom negotiation; social capital; and exposure to health information.

## Data Availability

The Henry M. Jackson Foundation for the Advancement of Military Medicine (HJF) and the U.S. Department of the Army are committed to safeguarding the privacy of all research participants. Due to the unique vulnerability of the Nigerian MSM and TGW participants in this study, the study investigators and ethical review committees have implemented additional measures to ensure participant anonymity is maintained in all reporting of research data. Distribution of de-identified participant-level data and accompanying research resources will require compliance with all applicable regulatory and ethical processes. Requests for these materials can be made via e-mail to PubRequest@hivresearch.org.

## Abbreviations

ART: Antiretroviral therapy
CI: Confidence interval
FSP: Female sexual partners
IQR: Interquartile range
IRB: Institutional review board
MSM: Men who have sex with men
MSP: Male sexual partners
PLWH: People living with HIV
RR: Risk ratio
TGW: Transgender women
