# Supplementary material for: HIV status disclosure by Nigerian men who have sex with men and transgender women living with HIV: a cross-sectional analysis at enrollment into an observational cohort
Source: BMC Public Health. 2020 Aug 26;20:1282. doi: 10.1186/s12889-020-09315-y (PMC7448976; doi:10.1186/s12889-020-09315-y)
Supplement: Supplementary file 1 — Additional file 1: Supplementary File 1. TRUST/RV368 Questionnaire (Visit 0). Participant demographics, perceptions and experiences of stigma, and sexual behaviors were evaluated using questionnaires developed specifically for this study that were administered by a trained interviewer over the first two study visits, approximately two weeks apart. The first set of questions (Visit 0) covered demographic and socioeconomic information; human rights and exposure to violations; disclosure of same-sex sexual practices; risk behavior with sexual partners; depression; and knowledge, attitudes, and behavior. [file 12889_2020_9315_MOESM1_ESM.pdf]

Draft

TRUST QUESTIONNAIRE

VISIT 0

Module 1 - Demographic and Socio Economic Information

Visit Date (dd/mm/yyyy) __ __ / __ __ / __ __ __ __	Interviewer ID __ __	Location: ○ CHC ○ 68 NARHY
Study Number:	RDS Coupon Number used to recruit participant	

"This first set of questions are about your background"

No.	Question	Coded Responses (Shade in the appropriate circles)
1.01	How old are you	*[Age in years]*<br>88 = Refusal<br>99 = Don't know
1.02	What is the highest level of school you attended: Quranic only, Primary, Junior secondary, Senior secondary, or Higher than Senior secondary?	○ 01 = Never attended school<br>○ 02 = Quranic only<br>○ 03 = Primary<br>○ 04 = Junior Secondary/JSS<br>○ 05 = Senior Secondary/SSS<br>○ 06 = Higher than SSS<br>○ 88 = Refusal<br>○ 99 = Don't know
1.03	What is your religion?	○ 01 = Protestant/Other Christian<br>○ 02 = Muslim<br>○ 03 = No religion<br>○ 04 = Other<br>○ 88 = Refusal<br>○ 99 = Don't know<br>Specify other: ________
1.04	What is the occupation from which you earn most of your income? (choose one)	○ 01 = Not Working (support from someone else)<br>○ 02 = Pupil/Student (support from someone else)<br>○ 03 = Professional<br>○ 04 = Self-employed business man<br>○ 05 = Entertainment/Service/Bar/Restaurant/Hotel<br>○ 06 = Driver/Laborer<br>○ 07 = Military/Police<br>○ 08 = Other
1.05	What was your income last month (in NGN)?	*[Record the number in box.]*<br>888888 = Refusal<br>999999 = Don't know
1.06	In which LGA do you currently live?	*[Select from the options at the bottom of the page]*<br>77 = Other (Specify) ________
1.07	For how many years have you lived in this same LGA?	*[Record Time in Years. If less than one year, record 00]*<br>90 = Entire Life<br>88 = Refusal<br>99 = Don't know
1.08	In the past 12 months, how many times have you been away from your home for one continuous month or more?	*[Record Number of times]*<br>88 = Refusal<br>99 = Don't know
1.12	In places where you stay currently, who stays there with you (mark all that apply)	
	1.12a = No one/lives alone	○ 01 = No<br>○ 02 = Yes<br>○ 88 = Refusal<br>○ 99 = Don't know

Lagos Local Government Areas (LGA)			Local Council Development Areas (LCDA)					
01 = Agege	09 = Ibeju-Lekki	17 = Ojo	21 = Agbado/Oke Odo	29 = Ejigbo	37 = Ikorodu West	45 = Itire /Ikate	53 = Onigbongbo
02 = Ajeromi Ifelodun	10 = Ifako Ijaiye	18 = Oshodi Isolo	22 = Agboyi-Ketu	30 = Eredo	38 = Ikosi Ejinrin	46 = Lagos Island East	54 = Oriade
03 = Alimosho	11 = Ikeja	19 = Shomolu	23 = Apapa-Iganmu	31 = Eti-Osa East	39 = Ikosi Isheri	47 = Lekki	55 = Orile Agege
04 = Apapa	12 = Ikorodu	20 = Surulere	24 = Ayobo-Ipaja	32 = Iba	40 = Igando Ikotun	48 = Mosan Okunola	56 = Oto Awori
05 = Amuwo-Odofin	13 = Kosofe		25 = Badagry West	33 = Ifelodun	41 = Ikoyi /Obalende	49 = Odi-Olowo	57 = Yaba
06 = Badagry	14 = Lagos Island		26 = Bariga	34 = Igbogbo/Baiyeku	42 = Imota	50 = Ojodu	
07 = Epe	15 = Lagos Mainland		27 = Coker Aguda	35 = Ijede	43 = Iru-Victoria Island	51 = Ojokoro	
08 = Eti-Osa West	16 = Mushin		28 = Egbe Idimu	36 = Ikorodu North	44 = Isolo	52 = Olorunda	

Study Number:

Visit Date (dd/mm/yyyy) __ __ / __ __ / __ __ __ __

No.	Question	Coded Responses (Shade in the appropriate circles)
	1.12b = Male sexual partner	○ 01 = No<br>○ 02 = Yes<br>○ 88 = Refusal<br>○ 99 = Don't know
	1.12c = Female sexual partner	○ 01 = No<br>○ 02 = Yes<br>○ 88 = Refusal<br>○ 99 = Don't know
	1.12d = Parents or other older relatives	○ 01 = No<br>○ 02 = Yes<br>○ 88 = Refusal<br>○ 99 = Don't know
	1.12e = Siblings or other family members who are similar to you in age	○ 01 = No<br>○ 02 = Yes<br>○ 88 = Refusal<br>○ 99 = Don't know
	1.12f = Friend(s)	○ 01 = No<br>○ 02 = Yes<br>○ 88 = Refusal<br>○ 99 = Don't know
	1.12g = Colleague(s)	○ 01 = No<br>○ 02 = Yes<br>○ 88 = Refusal<br>○ 99 = Don't know
	1.12h = Other	○ 01 = No<br>○ 02 = Yes (specify): ______<br>○ 88 = Refusal<br>○ 99 = Don't know
1.13	What do you consider your sexual orientation to be?	○ 01 = Gay or homosexual<br>○ 02 = Bisexual<br>○ 03 = Queer<br>○ 04 = Heterosexual or straight<br>○ 06 = Other<br>○ 88 = Refusal<br>○ 99 = Don't know<br>Specify other: ______
1.14	What do you consider your gender to be?	○ 01 = Man<br>○ 02 = Woman<br>○ 03 = Other (Specify): ______<br>○ 04 = Both male and female<br>○ 88 = Refusal<br>○ 99 = Don't know
1.15	What is your marital status?	○ 01 = Married to a woman<br>○ 02 = Cohabiting with a woman<br>○ 03 = Cohabitating with a man<br>○ 04 = Divorced/Separated<br>○ 05 = Widowed<br>○ 06 = Single/Never Married<br>○ 07 = Other<br>○ 88 = Refusal<br>○ 99 = Don't know<br>Specify other: ______
1.17	How many children do you have?	*[All numbers]*<br>88 = Refusal<br>99 = Don't Know<br>☐☐
1.18	Do you currently own a mobile phone?	○ 01 = No<br>○ 02 = Yes<br>○ 88 = Refusal<br>○ 99 = Don't know

Module 1 - Demographic and Socio Economic Information

Study Number: ______

Visit Date (dd/mm/yyyy) __ __ / __ __ / __ __ __ __

No.	Question	Coded Responses (Shade in the appropriate circles)
	1.18a = Time from home to clinic?	88 = Refusal<br>99 = Don't Know<br>__ __ (hours) __ __ (minutes)
1.19	How often do you use the internet?	○ 01 = Never<br>○ 02 = Less than once a week<br>○ 03 = About once a week<br>○ 04 = 2 to 3 times per week<br>○ 05 = Almost every day<br>○ 88 = Refusal<br>○ 99 = Don't know

Module 2 - Human Rights and Exposure to Violations

"Now we are going to ask you questions about how you have felt or been treated by others because you have sex with men. Please do not feel bad about answering as there is no right or wrong answer, and we will not tell anyone about what you will tell us."

No.	Question	Coded Responses (Shade in the appropriate circles)
2.01	Have you ever felt excluded from family gatherings because you have sex with men?	○ 01 = No<br>○ 02 = Yes<br>○ 88 = Refusal<br>○ 99 = Don't know
2.02	Have you ever felt that family members have made discriminatory remarks or gossiped about you because you have sex with men?	○ 01 = No<br>○ 02 = Yes<br>○ 88 = Refusal<br>○ 99 = Don't know
2.03	Have you ever felt rejected by your friends because you have sex with men?	○ 01 = No<br>○ 02 = Yes<br>○ 88 = Refusal<br>○ 99 = Don't know
	HEALTH SYSTEM	
2.08	Have you ever felt afraid to go to health care services because you worry someone may learn you have sex with men?	○ 01 = No<br>○ 02 = Yes<br>○ 88 = Refusal<br>○ 99 = Don't know
2.09	Have you ever avoided going to health care services because you worry someone may learn you have sex with men?	○ 01 = No<br>○ 02 = Yes<br>○ 88 = Refusal<br>○ 99 = Don't know
2.10	Have you ever been denied health services (or someone kept you from receiving health services) because you have sex with men?	○ 01 = No<br>○ 02 = Yes<br>○ 88 = Refusal<br>○ 99 = Don't know
2.11	Have you ever felt that you were not treated well in a health center because someone knew that you have sex with men?	○ 01 = No<br>○ 02 = Yes<br>○ 88 = Refusal<br>○ 99 = Don't know
2.13	Have you ever heard health care providers gossiping about you because you have sex with men?	○ 01 = No<br>○ 02 = Yes<br>○ 88 = Refusal<br>○ 99 = Don't know
	SOCIETY	
2.16	Have you ever felt that the police refused to protect you because you have sex with men?	○ 01 = No<br>○ 02 = Yes<br>○ 88 = Refusal<br>○ 99 = Don't know

Study Number:

Visit Date (dd/mm/yyyy) __ __ / __ __ / __ __ __ __

No.	Question	Coded Responses (Shade in the appropriate circles)
2.17	Have you ever been arrested?	◯ 01 = No [Skip to 2.25]<br>◯ 02 = Yes<br>◯ 88 = Refusal [Skip to 2.25]<br>◯ 99 = Don't know [Skip to 2.25]
	2.17a Were you arrested because you have sex with men?	◯ 01 = No<br>◯ 02 = Yes<br>◯ 88 = Refusal<br>◯ 99 = Don't know
2.25	Have you ever felt scared to walk around in public places because you have sex with men?	◯ 01 = No<br>◯ 02 = Yes<br>◯ 88 = Refusal<br>◯ 99 = Don't know
2.26	Have you ever been verbally harassed and felt it was because you have sex with men?	◯ 01 = No<br>◯ 02 = Yes<br>◯ 88 = Refusal<br>◯ 99 = Don't know
2.27	Have you ever been blackmailed by someone because you have sex with men?	◯ 01 = No<br>◯ 02 = Yes<br>◯ 88 = Refusal<br>◯ 99 = Don't know
2.28	Have you ever been pushed, shoved, slapped, hit, kicked, choked, or otherwise physically hurt by someone?	◯ 01 = No [skip to 2.35]<br>◯ 02 = Yes<br>◯ 88 = Refusal [skip to 2.35]<br>◯ 99 = Don't know [skip to 2.35]
	2.31 Who was the person who physically hurt you? I will read you the following options, and please tell me for each one whether this type of person ever beat you up or physically hurt you.	
	2.31a = Wife, girlfriend, or any current or past female sexual partner	◯ 01 = No<br>◯ 02 = Yes<br>◯ 88 = Refusal<br>◯ 99 = Don't know
	2.31b = Boyfriend or any current or past male sexual partner	◯ 01 = No<br>◯ 02 = Yes<br>◯ 88 = Refusal<br>◯ 99 = Don't know
	2.32 Do you believe any of these experiences of physical violence was/were related to the fact that you have sex with men?	◯ 01 = No<br>◯ 02 = Yes<br>◯ 88 = Refusal<br>◯ 99 = Don't know
2.35	Have you ever been forced to have sex when you did not want to? (By forced, I mean physically forced, coerced to have sex, or penetrated with an object, when you did not want to).	◯ 01 = No [skip to Module 3]<br>◯ 02 = Yes<br>◯ 88 = Refusal [skip to Module 3]<br>◯ 99 = Don't know [skip to Module 3]
	2.36 Approximately, how many times have you been forced to have sex when you did not want to?	*[Record the number of times]*<br>88 = Refusal<br>99 = Don't Know<br>__ __
	2.37 How would you classify the person(s) who forced you to have sex? I will read you the following options, and please tell me for each one whether this type of person ever forced you to have sex when you did not want to.	
	2.37a = Wife, girlfriend, or any current or past female sexual partner	◯ 01 = No ◯ 88 = Refusal<br>◯ 02 = Yes ◯ 99 = Don't know

Study Number: ______ Visit Date (dd/mm/yyyy) __ / __ / ____

No.	Question	Coded Responses (Shade in the appropriate circles)
	2.37b = Boyfriend or any current or past male sexual partner	○ 01 = No<br>○ 02 = Yes<br>○ 88 = Refusal<br>○ 99 = Don't know
	2.38 Do you believe any of these experiences of sexual violence were related to the fact that you have sex with men?	○ 01 = No<br>○ 02 = Yes<br>○ 88 = Refusal<br>○ 99 = Don't know
	2.39 Approximately, how old were you the first time you were forced to have sex when you did not want to? (age in years)	*[Record age in years]*<br>88 = Refusal<br>99 = Don't Know<br>☐☐
	2.40 After someone forced you to have sex, who did you tell about this experience(s)? I will read you the following options, and please tell me for each one whether you told this person about the experience(s).	
	2.40a = Nobody	○ 01 = No<br>○ 02 = Yes [Skip to 2.41]<br>○ 88 = Refusal<br>○ 99 = Don't know
	2.40b = Wife, girlfriend or female partner	○ 01 = No<br>○ 02 = Yes<br>○ 88 = Refusal<br>○ 99 = Don't know
	2.40c = Boyfriend or partner	○ 01 = No<br>○ 02 = Yes<br>○ 88 = Refusal<br>○ 99 = Don't know
	2.41 Was the person who forced you to have sex ever arrested by the police for this?	○ 01 = No, none of the perpetrators were arrested<br>○ 02 = Yes, all of the perpetrators were arrested<br>○ 03 = Yes, but not all of the perpetrators<br>○ 04 = Other<br>○ 88 = Refusal<br>○ 99 = Don't know<br>Specify other: ______
	2.42 When was the last time you were forced to have sex against your will?	○ 01 = Within the last 12 months<br>○ 02 = More than 12 months ago<br>○ 88 = Refusal<br>○ 99 = Don't know

Module 3 - Disclosure

DISCLOSURE - Now I want to ask about people you may or may not have told about having sex with men or about being attracted to other men.

No.	Question	Coded Responses (Shade in the appropriate circles)
3.01	Have you told any member of your family that you have sex with other men or that you are attracted to other men?	○ 01 = No<br>○ 02 = Yes<br>○ 88 = Refusal<br>○ 99 = Don't know
3.02	Does anyone in your family know that you have sex with other men or that you are attracted to other men, including those you may not have told yourself?	○ 01 = No<br>○ 02 = Yes<br>○ 88 = Refusal<br>○ 99 = Don't know
3.03	Have you told any health care worker that you have sex with other men or that you are attracted to other men?	○ 01 = No<br>○ 02 = Yes<br>○ 88 = Refusal<br>○ 99 = Don't know

Study Number: ____________ Visit Date (dd/mm/yyyy) __ __ / __ __ / __ __ __ __

"I'd like to start by asking you about your sexual partners and behaviors. Please do not feel bad about answering as there is no right or wrong answer and we will do our best to ensure that no one finds out what you have told us. Unless otherwise specified, when I ask about condom use, I mean male or female condoms, it was a new condom that was used each time, and the condom was used for the entire penetrative sexual act. When I ask about sex with other men I mean anal sex unless otherwise specified. When I ask about anal sex, I mean either insertive ("top") or receptive ("bottom") unless otherwise specified. When I ask about oral sex, I mean giving or receiving."

No.	Question	Coded Responses (Shade in the appropriate circles)
4.01	What was the gender of the first person you ever had sex with (other than oral sex)?	○ 01 = Man ○ 88 = Refusal<br>○ 02 = Woman ○ 99 = Don't know
4.02	Approximately how old were you the first time you had anal sex with another man?	*[Record age in years]*<br>88 = Refusal<br>99 = Don't Know<br>☐☐
4.03	Who was the first man you ever had anal sex with?	○ 01 = Boyfriend ○ 05 = Stranger<br>○ 02 = Friend ○ 06 = Other<br>○ 03 = Colleague ○ 88 = Refusal<br>○ 04 = Family member ○ 99 = Don't know<br>Specify other: ____________
4.04	Have you ever had anal sex without a condom?	○ 01 = No<br>○ 02 = Yes<br>○ 88 = Refusal<br>○ 99 = Don't know
Now I want to ask you about your sexual experiences with men.		
4.05	Where (in what type of place) do you meet new male sexual partners? I will read you a list of possible places. For each one, please tell me whether you meet new male sexual partners there or not.	
	4.05a = Private Home	○ 01 = No<br>○ 02 = Yes<br>○ 88 = Refusal<br>○ 99 = Don't know
	4.05b = Bar or club	○ 01 = No<br>○ 02 = Yes<br>○ 88 = Refusal<br>○ 99 = Don't know
	4.05c = Private party	○ 01 = No<br>○ 02 = Yes<br>○ 88 = Refusal<br>○ 99 = Don't know
	4.05d = Brothel	○ 01 = No<br>○ 02 = Yes<br>○ 88 = Refusal<br>○ 99 = Don't know
	4.05e = Street or park	○ 01 = No<br>○ 02 = Yes<br>○ 88 = Refusal<br>○ 99 = Don't know
	4.05f = Private vehicle	○ 01 = No<br>○ 02 = Yes<br>○ 88 = Refusal<br>○ 99 = Don't know
	4.05g = Hotel or guest house	○ 01 = No<br>○ 02 = Yes<br>○ 88 = Refusal<br>○ 99 = Don't know

Study Number: ____________

Visit Date (dd/mm/yyyy) __ __ / __ __ / __ __ __ __

No.	Question	Coded Responses (Shade in the appropriate circles)
	4.05h = News advertisements or cards	○ 01 = No ○ 88 = Refusal<br>○ 02 = Yes ○ 99 = Don't know
	4.05i = Online	○ 01 = No ○ 88 = Refusal<br>○ 02 = Yes ○ 99 = Don't know
	4.05j = School or work	○ 01 = No ○ 88 = Refusal<br>○ 02 = Yes ○ 99 = Don't know
	4.05k = Mosque or church	○ 01 = No ○ 88 = Refusal<br>○ 02 = Yes ○ 99 = Don't know
	4.05l = Other (specify)	○ 01 = No<br>○ 02 = Yes (specify): ____________<br>○ 88 = Refusal<br>○ 99 = Don't know
Now I will ask you some questions about your experiences in the last 12 months with your male sexual partners.		
4.07	In the last 12 months, how many men did you have oral sex with?	*[Record the number of men. If 00 skip to 4.09]* ☐☐☐<br>888 = Refusal *[skip to 4.09]*<br>999 = Don't know *[skip to 4.09]*
	4.08 In the last 12 months, of the times you had oral sex with another man, how often was a condom used?	○ 01 = Never ○ 05 = Always<br>○ 02 = Almost never ○ 88 = Refusal<br>○ 03 = About half the time ○ 99 = Don't know<br>○ 04 = Almost always
4.09	In the last 12 months, how many men did you have anal sex with?	*[Record the number of men. If 000 skip to 4.32]* ☐☐☐<br>88 = Refusal *[skip to 4.32]*<br>99 = Don't know *[skip to 4.32]*
	4.09d In the last 12 months, did you only have anal sex with men who have the same HIV status as you?	○ 01 = No<br>○ 02 = Yes<br>○ 88 = Refusal<br>○ 99 = Don't know
	4.09e How often do you use condoms when you have anal sex with men of the same HIV status as you?	○ 01 = Never ○ 05 = Always<br>○ 02 = Almost never ○ 88 = Refusal<br>○ 03 = About half the time ○ 99 = Don't know<br>○ 04 = Almost always
	4.09f In the last 12 months, what type of anal sex do you engage in with your sexual partners?	○ 01 = Only insertive<br>○ 02 = Only receptive<br>○ 03 = Both insertive and receptive<br>○ 88 = Refusal<br>○ 99 = Don't know
4.10	Thinking of the times you have had anal sex with a man, was there a time when a condom slipped off or broke?	○ 01 = No [skip to 4.12] ○ 88 = Refusal [skip to 4.12]<br>○ 02 = Yes, in the last month ○ 99 = Don't know [skip to 4.12]<br>○ 03 = Yes, in the last 12 months<br>○ 04 = Yes, but not in the last 12 months
	4.11 When you have used a condom during anal sex with a man, how often has the condom slipped off or broken?	○ 01 = Never ○ 05 = Always<br>○ 02 = Almost never ○ 88 = Refusal<br>○ 03 = About half the time ○ 99 = Don't know<br>○ 04 = Almost always

Module 4 - RISK BEHAVIOR - A. PARTNERS

Study Number: ____________

Visit Date (dd/mm/yyyy) __ __ / __ __ / __ __ __ __

No.	Question	Coded Responses (Shade in the appropriate circles)
4.12	In the last 12 months, how many men did you have insertive anal sex with? [Insertive sex means anal sex when YOU inserted your penis into your partner's anus. Some people call this being 'the top']	*[Record the number of men. If 00 skip to 4.15]* ☐☐☐<br>888 = Refusal *[skip to 4.15]*<br>999 = Don't know *[skip to 4.15]*
	4.13 In the last 12 months, of the times you had insertive anal sex with a man, how often were condoms used?	○ 01 = Never [skip to 4.15]<br>○ 02 = Almost never<br>○ 03 = About half the time<br>○ 04 = Almost always<br>○ 05 = Always<br>○ 88 = Refusal [skip to 4.15]<br>○ 99 = Don't know [skip to 4.15]
4.15	In the last 12 months, how many men did you have receptive anal sex with? [Receptive sex means anal sex when your partner inserted HIS penis into your anus. Some people call this being 'the bottom']	*[Record the number of men. If 00 skip to 4.30]* ☐☐☐<br>888 = Refusal *[skip to 4.30]*<br>999 = Don't know *[skip to 4.30]*
	4.16 In the last 12 months, of the times you had receptive anal sex with a man, how often were condoms used?	○ 01 = Never<br>○ 02 = Almost never<br>○ 03 = About half the time<br>○ 04 = Almost always<br>○ 05 = Always<br>○ 88 = Refusal<br>○ 99 = Don't know
4.18	In the last 12 months, of the men you've had anal sex with (either insertive or receptive), how many of them were main partners? By main partner, I mean a man that you have sex with and with whom you feel committed to. This means a partner that you would call your boyfriend, significant other, or life partner.	*[Record the number of men. If 00 skip to 4.24]* ☐☐☐<br>888 = Refusal *[skip to 4.24]*<br>999 = Don't know *[skip to 4.24]*
	4.19 In the last 12 months, have you talked with your main male sexual partner(s) about sexually transmitted infections and HIV?	○ 01 = No<br>○ 02 = With some but not all main male partners<br>○ 03 = Yes, with all main male partners (or with the only one)<br>○ 88 = Refusal<br>○ 99 = Don't know
	4.20 In the last 12 months, have you talked with your main male sexual partner(s) about their HIV status? (By this, I mean you ask or they tell you they have been tested and tell you the results, regardless of whether positive or negative)?	○ 01 = No<br>○ 02 = With some but not all main male partners<br>○ 03 = Yes, with all main male partners (or with the only one)<br>○ 88 = Refusal<br>○ 99 = Don't know
	4.21 In the last 12 months, have you talked with your main male sexual partner(s) about your HIV status? (By this, I mean they ask or you tell them you have been tested and tell them the results, regardless of whether positive or negative)	○ 01 = No<br>○ 02 = With some but not all main male partners<br>○ 03 = Yes, with all main male partners (or with the only one)<br>○ 88 = Refusal<br>○ 99 = Don't know
	4.22 Was a condom used the last time that you had anal sex with your main male partner(s)?	○ 01 = No<br>○ 02 = Yes<br>○ 88 = Refusal<br>○ 99 = Don't know
	4.23 How often are condoms used when you have anal sex with men who are your main male partner(s)?	○ 01 = Never<br>○ 02 = Almost never<br>○ 03 = About half the time<br>○ 04 = Almost always<br>○ 05 = Always<br>○ 88 = Refusal<br>○ 99 = Don't know
4.24	Of the men you've had anal sex with in the last 12 months, approximately how many of them were casual partners? By casual partner, this means a man that you have sex with, but you don't feel committed to.	*[Record the number of men. If 000 skip to 4.30]* ☐☐☐<br>888 = Refusal *[skip to 4.30]*<br>999 = Don't know *[skip to 4.30]*
	4.25 In the last 12 months, have you talked with your casual male sexual partner(s) about sexually transmitted infections and HIV?	○ 01 = No<br>○ 02 = With some but not all casual male partners<br>○ 03 = Yes, with all casual male partners (or with the only one)<br>○ 88 = Refusal<br>○ 99 = Don't know

Study Number: ______

Visit Date (dd/mm/yyyy) __ __ / __ __ / __ __ __ __

No.	Question	Coded Responses (Shade in the appropriate circles)
	4.26 In the last 12 months, have you talked with your casual male sexual partner(s) about their HIV status?<br>*(By this, I mean you ask or they tell you they have been tested and tell you the results, regardless of whether positive or negative)?*	○ 01 = No<br>○ 02 = With some but not all casual male partners<br>○ 03 = Yes, with all casual male partners (or with the only one)<br>○ 88 = Refusal<br>○ 99 = Don't know
	4.27 In the last 12 months, have you talked with your casual male sexual partner(s) about your HIV status?<br>*(By this, I mean they ask or you tell them you have been tested and tell them the results, regardless of whether positive or negative)*	○ 01 = No<br>○ 02 = With some but not all casual male partners<br>○ 03 = Yes, with all casual male partners (or with the only one)<br>○ 88 = Refusal<br>○ 99 = Don't know
	4.28 Was a condom used the last time that you had anal sex with a man who was a casual partner?	○ 01 = No<br>○ 02 = Yes<br>○ 88 = Refusal<br>○ 99 = Don't know
	4.29 How often are condoms used when you have anal sex with men who are casual partners?	○ 01 = Never<br>○ 02 = Almost never<br>○ 03 = About half the time<br>○ 04 = Almost always<br>○ 05 = Always<br>○ 88 = Refusal<br>○ 99 = Don't know
4.30	Thinking about when you had sex with any men in the last 12 months, how many men did you have anal or oral sex with in exchange for things you wanted or needed such as money, drugs, food, shelter or transportation?	*[Record the number of men. If 000, skip to 4.31]* __ __ __<br>888 = Refusal *[skip to 4.31]*<br>999 = Don't know *[skip to 4.31]*
	4.30a Were you paid money?	○ 01 = No<br>○ 02 = Yes<br>○ 88 = Refusal<br>○ 99 = Don't know
	4.30b How would you classify the men who <u>gave you</u> money or things you needed for anal or oral sex?	○ 01 = Boyfriend<br>○ 02 = Friend<br>○ 03 = Colleague<br>○ 04= Family member<br>○ 05 = Stranger<br>○ 06 = Other<br>○ 88 = Refusal<br>○ 99 = Don't know<br>(specify): ______
4.31	Thinking about when you had sex with any men in the last 12 months, how many men did you have anal or oral sex with in exchange for things they wanted or needed such as money, drugs, food, shelter or transportation?	*[Record the number of men. If 000, skip to 4.32]* __ __ __<br>888 = Refusal *[skip to 4.32]*<br>999 = Don't know *[skip to 4.32]*
	4.31a Did you pay money?	○ 01 = No<br>○ 02 = Yes<br>○ 88 = Refusal<br>○ 99 = Don't know
	4.31b How would you classify the men who <u>you gave</u> money or things they needed for anal or oral sex?	○ 01 = Boyfriend<br>○ 02 = Friend<br>○ 03 = Colleague<br>○ 04= Family member<br>○ 05 = Stranger<br>○ 06 = Other<br>○ 88 = Refusal<br>○ 99 = Don't know<br>(specify): ______
Now I want to ask you about your sexual experiences with men in the last month		
4.32	In the last month approximately how many male partners did you have any type of sex with?	*[Record the number of male partners]* __ __<br>88 = Refusal<br>99 = Don't know

Study Number:

Visit Date (dd/mm/yyyy) __ __ / __ __ / __ __ __ __

No.	Question	Coded Responses (Shade in the appropriate circles)
4.33	In the last month approximately how many male partners did you have anal sex with?	*[Record the number of male partners]*<br>88 = Refusal<br>99 = Don't know
4.34	In the last month how often were condoms used when you had anal sex with male partners?	◯ 01 = Never<br>◯ 02 = Almost never<br>◯ 03 = About half the time<br>◯ 04 = Almost always<br>◯ 05 = Always<br>◯ 88 = Refusal<br>◯ 99 = Don't know
4.35	Now think about the last time you had anal sex with a male partner. Was a condom used at that time?	◯ 01 = No<br>◯ 02 = Yes<br>◯ 88 = Refusal<br>◯ 99 = Don't know
4.36	Now think about the last time a condom was not used with a male partner during anal sex. Why wasn't a condom used at that time? I will read some possible reasons, and for each one please tell me whether that reason applies to your situation or not.	
	4.36b = We forgot or didn't think about it	◯ 01 = No<br>◯ 02 = Yes<br>◯ 88 = Refusal<br>◯ 99 = Don't know
	4.36c = There were no condoms available	◯ 01 = No<br>◯ 02 = Yes<br>◯ 88 = Refusal<br>◯ 99 = Don't know
	4.36d = There was no time to find a condom	◯ 01 = No<br>◯ 02 = Yes<br>◯ 88 = Refusal<br>◯ 99 = Don't know
	4.36e = I don't like using condoms	◯ 01 = No<br>◯ 02 = Yes<br>◯ 88 = Refusal<br>◯ 99 = Don't know
	4.36g = I do not feel comfortable buying condoms	◯ 01 = No<br>◯ 02 = Yes<br>◯ 88 = Refusal<br>◯ 99 = Don't know
	4.36h = Condoms are not necessary	◯ 01 = No<br>◯ 02 = Yes<br>◯ 88 = Refusal<br>◯ 99 = Don't know
	4.36i = I don't trust condoms	◯ 01 = No<br>◯ 02 = Yes<br>◯ 88 = Refusal<br>◯ 99 = Don't know
	4.36j = I used another method to prevent HIV or sexually transmitted infection (specify method)	◯ 01 = No<br>◯ 02 = Yes (specify): ________________<br>◯ 88 = Refusal<br>◯ 99 = Don't know
	4.36k = If I used or suggested using a condom, my partner would think I have an infection	◯ 01 = No<br>◯ 02 = Yes<br>◯ 88 = Refusal<br>◯ 99 = Don't know

Study Number: ____________ Visit Date (dd/mm/yyyy) __ __ / __ __ / __ __ __ __

No.	Question	Coded Responses (Shade in the appropriate circles)
	4.36l = My partner doesn't like them or objected	○ 01 = No<br>○ 02 = Yes<br>○ 88 = Refusal<br>○ 99 = Don't know
	4.36m = My partner is/was a safe partner	○ 01 = No<br>○ 02 = Yes<br>○ 88 = Refusal<br>○ 99 = Don't know
	4.36n = My partner removed the condom	○ 01 = No<br>○ 02 = Yes<br>○ 88 = Refusal<br>○ 99 = Don't know
	4.36q = The sex was forced	○ 01 = No<br>○ 02 = Yes<br>○ 88 = Refusal<br>○ 99 = Don't know
	4.36s = Other (specify)	○ 01 = No<br>○ 02 = Yes (specify): ____________<br>○ 88 = Refusal<br>○ 99 = Don't know
4.37	Counting all of your male partners, in an average week, approximately how many times do you have anal sex with a man?	*[Record the number of sex acts]* ☐☐<br>88 = Refusal<br>99 = Don't know

Module 4B: FEMALE PARTNERS

"Next I am going to ask you about your female sexual partners."

No.	Question	Coded Responses (Shade in the appropriate circles)
4.38	Have you ever had any type of sex with a woman?	○ 01 = No [skip to 4.74]<br>○ 02 = Yes, within the past 5 years<br>○ 03 = Yes, but not in the past 5 years<br>○ 88 = Refusal [skip to 4.74]<br>○ 99 = Don't know [skip to 4.74]
4.39	Approximately how old were you the first time you had any type of sex with a woman?	*[Record age in years]* ☐☐<br>88 = Refusal<br>99 = Don't know
4.40	Who was the first woman you ever had any type of sex with?	○ 01 = Girlfriend<br>○ 02 = Friend<br>○ 03 = Colleague<br>○ 04= Family member<br>○ 05 = Stranger<br>○ 06 = Other<br>○ 88 = Refusal<br>○ 99 = Don't know<br>(specify): ____________
	Now I will ask you some questions about your experiences in the last 12 months with your female sexual partners.	
4.43	Thinking of the times you have had vaginal or anal sex with a woman, was there a time when a condom slipped off or broke?	○ 01 = No<br>○ 02 = Yes, In the last month<br>○ 03 = Yes, in the last 12 months<br>○ 04 = Yes, but not in the last 12 months<br>○ 88 = Refusal<br>○ 99 = Don't know
	4.44 When you have used a condom during vaginal or anal sex with a woman, how often has the condom slipped off or broken?	○ 01 = Never<br>○ 02 = Almost never<br>○ 03 = About half the time<br>○ 04 = Almost always<br>○ 05 = Always<br>○ 88 = Refusal<br>○ 99 = Don't know

Study Number:

Visit Date (dd/mm/yyyy) / /

No.	Question	Coded Responses (Shade in the appropriate circles)
4.45	In the last 12 months, how many women did you have oral sex with?	*[Record the number of women. If 000 skip to 4.47]*<br>888 = Refusal [skip to 4.47]<br>999 = Don't know [skip to 4.47]
	4.46 Of the times you had oral sex with a woman in the last 12 months, how often was a condom used?	◯ 01 = Never<br>◯ 02 = Almost never<br>◯ 03 = About half the time<br>◯ 04 = Almost always<br>◯ 05 = Always<br>◯ 88 = Refusal<br>◯ 99 = Don't know
4.47	In the last 12 months, how many women did you have vaginal sex with?	*[Record the number of women. If 000 skip to 4.50]*<br>888 = Refusal [skip to 4.50]<br>999 = Don't know [skip to 4.50]
	4.48 Of the times you had vaginal sex with a woman in the last 12 months, how often was a condom used?	◯ 01 = Never<br>◯ 02 = Almost never<br>◯ 03 = About half the time<br>◯ 04 = Almost always<br>◯ 05 = Always<br>◯ 88 = Refusal<br>◯ 99 = Don't know
4.50	In the last 12 months, how many women did you have anal sex with?	*[Record the number of women. If 000 skip to 4.67]*<br>888 = Refusal [skip to 4.67]<br>999 = Don't know [skip to 4.67]
	4.51 Of the times you had anal sex with a woman in the last 12 months, how often was a condom used?	◯ 01 = Never<br>◯ 02 = Almost never<br>◯ 03 = About half the time<br>◯ 04 = Almost always<br>◯ 05 = Always<br>◯ 88 = Refusal<br>◯ 99 = Don't know
4.53	Of the women you've had anal or vaginal sex with in the last 12 months, how many of them were main partners?<br>*By main partner, I mean a woman that you have sex with and with whom you feel committed to. This is a partner that you would call your girlfriend, spouse, significant other, or life partner (You can list main female partners even if you listed main male partners).*	*[Record the number of women. If 000 skip to 4.59]*<br>888= Refusal [skip to 4.59]<br>999= Don't know [skip to 4.59]
	4.54 In the last 12 months, have you talked with your main female sexual partner(s) about sexually transmitted infections and HIV?	◯ 01 = No<br>◯ 02 = With some but not all main female partners<br>◯ 03 = Yes, with all main female partners (or with the only one)<br>◯ 88 = Refusal<br>◯ 99 = Don't know
	4.55 In the last 12 months, have you talked with your main female sexual partner(s) about their HIV status?<br>*(By this, I mean you ask or they tell you they have been tested and tell you the results, regardless of whether positive or negative)?*	◯ 01 = No<br>◯ 02 = With some but not all main female partners<br>◯ 03 = Yes, with all main female partners (or with the only one)<br>◯ 88 = Refusal<br>◯ 99 = Don't know
	4.56 In the last 12 months, have you talked with your main female sexual partner(s) about your HIV status?<br>*(By this, I mean they ask or you tell them you have been tested and tell them the results, regardless of whether positive or negative)*	◯ 01 = No<br>◯ 02 = With some but not all main female partners<br>◯ 03 = Yes, with all main female partners (or with the only one)<br>◯ 88 = Refusal<br>◯ 99 = Don't know
	4.57 Was a condom used the last time that you had vaginal or anal sex with a woman who you consider to be a main partner?	◯ 01 = No<br>◯ 02 = Yes<br>◯ 88 = Refusal<br>◯ 99 = Don't know
	4.58 How often are condoms used when you have vaginal or anal sex with women who you consider to be main partners?	◯ 01 = Never<br>◯ 02 = Almost never<br>◯ 03 = About half the time<br>◯ 04 = Almost always<br>◯ 05 = Always<br>◯ 88 = Refusal<br>◯ 99 = Don't know

Study Number: ____________

Visit Date (dd/mm/yyyy) __ __ / __ __ / __ __ __ __

No.	Question	Coded Responses (Shade in the appropriate circles)
4.59	Of the women you've had vaginal or anal sex with in the last 12 months, how many of them were casual partners?<br><br>*By casual partner, this means a woman that you have sex with, but you don't know very well or don't feel committed to.*	*[Record the number of women. If 000 skip to 4.65]*<br><br>888 = Refusal [skip to 4.65]<br>999 = Don't know [skip to 4.65]<br>☐☐☐
	4.60 In the last 12 months, have you talked with your casual female sexual partner(s) about sexually transmitted infections and HIV?	○ 01 = No<br>○ 02 = With some but not all casual female partners<br>○ 03 = Yes, with all casual female partners (or with the only one)<br>○ 88 = Refusal<br>○ 99 = Don't know
	4.61 In the last 12 months, have you talked with your casual female sexual partner(s) about their HIV status?<br><br>*(By this, I mean you ask or they tell you they have been tested and tell you the results, regardless of whether positive or negative)?*	○ 01 = No<br>○ 02 = With some but not all casual female partners<br>○ 03 = Yes, with all casual female partners (or with the only one)<br>○ 88 = Refusal<br>○ 99 = Don't know
	4.62 In the last 12 months, have you talked with your casual female sexual partner(s) about your HIV status?<br><br>*(By this, I mean they ask or you tell them you have been tested and tell them the results, regardless of whether positive or negative)*	○ 01 = No<br>○ 02 = With some but not all casual female partners<br>○ 03 = Yes, with all casual female partners (or with the only one)<br>○ 88 = Refusal<br>○ 99 = Don't know
	4.63 Was a condom used the last time that you had vaginal or anal sex with a woman who you consider to be a casual partner?	○ 01 = No<br>○ 02 = Yes<br><br>○ 88 = Refusal<br>○ 99 = Don't know
	4.64 How often are condoms used when you have vaginal or anal sex with women who are casual partners?	○ 01 = Never ○ 05 = Always<br>○ 02 = Almost never ○ 88 = Refusal<br>○ 03 = About half the time ○ 99 = Don't know<br>○ 04 = Almost always
4.65	Thinking about when you had sex with any woman in the last 12 months, how many women did you have vaginal, anal or oral sex with in exchange for things you wanted or needed, such as money, drugs, food, shelter or transportation?	*[Record the number of women. If 000 skip to 4.66]*<br><br>888 = Refusal [skip to 4.66]<br>999 = Don't know [skip to 4.66]<br>☐☐☐
	4.65a Were you paid money?	○ 01 = No<br>○ 02 = Yes<br><br>○ 88 = Refusal<br>○ 99 = Don't know
	4.65b How would you classify the women who <u>gave you</u> money or things you needed for vaginal, anal or oral sex?	○ 01 = Girlfriend ○ 05 = Stranger<br>○ 02 = Friend ○ 06 = Other<br>○ 03 = Colleague ○ 88 = Refusal<br>○ 04= Family member ○ 99 = Don't know<br><br>(specify): ____________
4.66	Thinking about when you had sex with any women in the last 12 months, how many women did you have vaginal, anal or oral sex with in exchange for things they wanted or needed like money, drugs, food, shelter or transportation?	*[Record the number of women. If 000 skip to 4.67]*<br><br>888 = Refusal [skip to 4.67]<br>999 = Don't know [skip to 4.67]<br>☐☐☐
	4.66a Did you pay money?	○ 01 = No<br>○ 02 = Yes<br><br>○ 88 = Refusal<br>○ 99 = Don't know

Study Number: ____________

Visit Date (dd/mm/yyyy) __ __ / __ __ / __ __ __ __

No.	Question	Coded Responses (Shade in the appropriate circles)
	4.66b How would you classify the women who <u>you gave</u> money or things they needed for vaginal, anal or oral sex?	○ 01 = Girlfriend<br>○ 02 = Friend<br>○ 03 = Colleague<br>○ 04= Family member<br>○ 05 = Stranger<br>○ 06 = Other<br>○ 88 = Refusal<br>○ 99 = Don't know<br>(If other, specify): ____________
	Now I want to ask you about your sexual experiences with women in the last month	
4.67	In the last month, approximately how many women did you have any type of sex with?	*[Record the number of female partners. If 00, skip to 4.72]*<br>88 = Refusal<br>99 = Don't know<br>☐☐
4.68	In the last month, approximately how many women did you have vaginal sex with?	*[Record the number of female partners. If 00, skip to 4.70]*<br>88 = Refusal<br>99 = Don't know<br>☐☐
	4.69 In the last month, how often were condoms used when you had vaginal sex with female partners?	○ 01 = Never<br>○ 02 = Almost never<br>○ 03 = About half the time<br>○ 04 = Almost always<br>○ 05 = Always<br>○ 88 = Refusal<br>○ 99 = Don't know
4.70	In the last month, approximately how many women did you have anal sex with?	*[Record the number of female partners. If 00, skip to 4.72]*<br>88 = Refusal<br>99 = Don't know<br>☐☐
	4.71 In the last month, how often were condoms used when you had anal sex with female partners?	○ 01 = Never<br>○ 02 = Almost never<br>○ 03 = About half the time<br>○ 04 = Almost always<br>○ 05 = Always<br>○ 88 = Refusal<br>○ 99 = Don't know
4.72	Now think about the last time you had vaginal or anal sex with a female partner. Was a condom used at that time?	○ 01 = No<br>○ 02 = Yes<br>○ 88 = Refusal<br>○ 99 = Don't know
4.73	Now think about the last time a condom was not used with a female partner during vaginal or anal sex. Why wasn't a condom used at that time? I will read some possible reasons, and for each one please tell me whether that reason applies to your situation or not.	
	4.73b = We forgot or didn't think about it	○ 01 = No ○ 88 = Refusal<br>○ 02 = Yes ○ 99 = Don't know
	4.73c = There were no condoms available	○ 01 = No ○ 88 = Refusal<br>○ 02 = Yes ○ 99 = Don't know
	4.73d = There was no time to find a condom	○ 01 = No ○ 88 = Refusal<br>○ 02 = Yes ○ 99 = Don't know
	4.73e = I don't like using condoms	○ 01 = No ○ 88 = Refusal<br>○ 02 = Yes ○ 99 = Don't know
	4.73g = I do not feel comfortable buying condoms	○ 01 = No ○ 88 = Refusal<br>○ 02 = Yes ○ 99 = Don't know

Study Number:

Visit Date (dd/mm/yyyy) __ __ / __ __ / __ __ __ __

No.	Question	Coded Responses (Shade in the appropriate circles)
	4.73h = Condoms are not necessary	○ 01 = No<br>○ 02 = Yes<br><br>○ 88 = Refusal<br>○ 99 = Don't know
	4.73i = I don't trust condoms	○ 01 = No<br>○ 02 = Yes<br><br>○ 88 = Refusal<br>○ 99 = Don't know
	4.73j = I used another method to prevent HIV or sexually transmitted infection	○ 01 = No<br>○ 02 = Yes (specify): ______<br><br>○ 88 = Refusal<br>○ 99 = Don't know
	4.73k = If I used a condom, my partner would think I have an infection	○ 01 = No<br>○ 02 = Yes<br><br>○ 88 = Refusal<br>○ 99 = Don't know
	4.73l = My partner doesn't like them or objected	○ 01 = No<br>○ 02 = Yes<br><br>○ 88 = Refusal<br>○ 99 = Don't know
	4.73m = My partner is/was a safe partner	○ 01 = No<br>○ 02 = Yes<br><br>○ 88 = Refusal<br>○ 99 = Don't know
	4.73r = I was trying to get my partner pregnant	○ 01 = No<br>○ 02 = Yes<br><br>○ 88 = Refusal<br>○ 99 = Don't know
	4.73s = My partner used another birth control method	○ 01 = No<br>○ 02 = Yes<br><br>○ 88 = Refusal<br>○ 99 = Don't know
	4.73t = Other (specify)	○ 01 = No<br>○ 02 = Yes (specify): ______<br><br>○ 88 = Refusal<br>○ 99 = Don't know
4.74	In the last 12 months, was there any time when you had two regular sexual partners at the same time; that is, two ongoing sexual partnerships, either with males or females? I will read possible types of ongoing sexual partnerships. For each one, please tell me if you had this combination of partners in the last 6 months.	
	4.74a = Male and female partners	○ 01 = No ○ 88 = Refusal<br>○ 02 = Yes ○ 99 = Don't know
	4.74b = Two or more male partners	○ 01 = No ○ 88 = Refusal<br>○ 02 = Yes ○ 99 = Don't know
	4.74c = Two or more female partners	○ 01 = No ○ 88 = Refusal<br>○ 02 = Yes ○ 99 = Don't know

Study Number:

Visit Date (dd/mm/yyyy) __ __ / __ __ / __ __ __ __

No.	Question	Coded Responses (Shade in the appropriate circles)
	4.74d = No concurrent partnerships in last 12 months	◯ 01 = No<br>◯ 02 = Yes<br>◯ 88 = Refusal<br>◯ 99 = Don't know
4.75	Do you currently have two or more regular sexual partners; that is, two or more ongoing sexual partnerships, either with males or females? I will read possible types of ongoing sexual partnerships. For each one, please tell me whether you currently have this combination of partners.	
	4.75a = Male and female partners	◯ 01 = No<br>◯ 02 = Yes<br>◯ 88 = Refusal<br>◯ 99 = Don't know
	4.75b = Two or more male partners	◯ 01 = No<br>◯ 02 = Yes<br>◯ 88 = Refusal<br>◯ 99 = Don't know
	4.75c = Two or more female partners	◯ 01 = No<br>◯ 02 = Yes<br>◯ 88 = Refusal<br>◯ 99 = Don't know
	4.75d = No concurrent partnerships in last 12 months	◯ 01 = No<br>◯ 02 = Yes<br>◯ 88 = Refusal<br>◯ 99 = Don't know
4.76	As far as you know, does/do your current partner(s) currently have ongoing sexual partnerships with other male or female partners?	◯ 01 = No<br>◯ 02 = Yes<br>◯ 88 = Refusal<br>◯ 99 = Don't know

Module 5 - Depression

"Now we are going to ask questions about your feelings of sadness or depression. Over the last 2 weeks, how often have you been bothered by any of the following problems? Please do not feel bad about answering as we will not tell anyone about what you tell us."

No.	Question	Coded Responses (Shade in the appropriate circles)
	5.07 Little interest or pleasure in doing things?	◯ 00 = Not at all ◯ 03 = Nearly every day<br>◯ 01 = Several days ◯ 88 = Refusal<br>◯ 02 = More than half the days ◯ 99 = Don't know
	5.08 Feeling down, depressed (sad), or hopeless?	◯ 00 = Not at all ◯ 03 = Nearly every day<br>◯ 01 = Several days ◯ 88 = Refusal<br>◯ 02 = More than half the days ◯ 99 = Don't know
	5.09 Either trouble falling or staying asleep, OR sleeping too much?	◯ 00 = Not at all ◯ 03 = Nearly every day<br>◯ 01 = Several days ◯ 88 = Refusal<br>◯ 02 = More than half the days ◯ 99 = Don't know
	5.10 Feeling tired or having little energy?	◯ 00 = Not at all ◯ 03 = Nearly every day<br>◯ 01 = Several days ◯ 88 = Refusal<br>◯ 02 = More than half the days ◯ 99 = Don't know
	5.11 Either poor appetite OR overeating	◯ 00 = Not at all ◯ 03 = Nearly every day<br>◯ 01 = Several days ◯ 88 = Refusal<br>◯ 02 = More than half the days ◯ 99 = Don't know

Module 5 - DEPRESSION

Study Number:

Visit Date (dd/mm/yyyy) __ __ / __ __ / __ __ __ __

No.	Question	Coded Responses (Shade in the appropriate circles)
	5.12 Feeling bad about yourself- or that you are a failure or have let yourself or your family down?	◯ 00 = Not at all ◯ 03 = Nearly every day<br>◯ 01 = Several days ◯ 88 = Refusal<br>◯ 02 = More than half the days ◯ 99 = Don't know
	5.13 Trouble concentrating on things, such as reading the newspaper or watching TV?	◯ 00 = Not at all ◯ 03 = Nearly every day<br>◯ 01 = Several days ◯ 88 = Refusal<br>◯ 02 = More than half the days ◯ 99 = Don't know
	5.14 Either moving or speaking so slowly that other people could have noticed OR being so fidgety or restless that you have been moving around a lot more than usual?	◯ 00 = Not at all ◯ 03 = Nearly every day<br>◯ 01 = Several days ◯ 88 = Refusal<br>◯ 02 = More than half the days ◯ 99 = Don't know
	5.15 Thought that you would be better off dead, or hurting yourself in some way?	◯ 00 = Not at all ◯ 03 = Nearly every day<br>◯ 01 = Several days ◯ 88 = Refusal<br>◯ 02 = More than half the days ◯ 99 = Don't know

Module 6 - Knowledge, Attitudes, Behavior

"Now we are going to ask you private questions about HIV prevention and sexual practices. Please do not feel bad about answering as it will remain confidential"

No.	Question	Coded Responses (Shade in the appropriate circles)
6.01	In the last 12 months, have you been tested for a sexually transmitted infection (this could include syphilis, gonorrhea, Chlamydia, herpes or other infection)?	◯ 01 = No<br>◯ 02 = Yes<br>◯ 88 = Refusal<br>◯ 99 = Don't know
6.02	In the last 12 months, have you been told by a doctor or other healthcare provider that you have a sexually transmitted infection (such as syphilis, gonorrhea or chlamydia, or herpes)? I will read a list of possible infections. For each one please tell me if you have been told by a doctor or other healthcare provider that you have this infection.	
	6.02a = Syphilis	◯ 01 = No<br>◯ 02 = Yes<br>◯ 88 = Refusal<br>◯ 99 = Don't know
	6.02b = Gonorrhea	◯ 01 = No<br>◯ 02 = Yes<br>◯ 88 = Refusal<br>◯ 99 = Don't know
	6.02c = Chlamydia	◯ 01 = No<br>◯ 02 = Yes<br>◯ 88 = Refusal<br>◯ 99 = Don't know
	6.02d = Herpes	◯ 01 = No<br>◯ 02 = Yes<br>◯ 88 = Refusal<br>◯ 99 = Don't know
	6.02e = Human Papillomavirus (HPV)	◯ 01 = No<br>◯ 02 = Yes<br>◯ 88 = Refusal<br>◯ 99 = Don't know
	6.02f = Hepatitis B	◯ 01 = No<br>◯ 02 = Yes<br>◯ 88 = Refusal<br>◯ 99 = Don't know

Study Number: ____________ Visit Date (dd/mm/yyyy) __ __ / __ __ / __ __ __ __

No.	Question	Coded Responses (Shade in the appropriate circles)
	6.02g = Hepatitis C	◯ 01 = No ◯ 88 = Refusal<br>◯ 02 = Yes ◯ 99 = Don't know
	6.02h = Other (specify)	◯ 01 = No<br>◯ 02 = Yes (specify): ____________<br>◯ 88 = Refusal<br>◯ 99 = Don't know
	6.03 Have you ever received a vaccine to prevent getting Hepatitis B infection?	◯ 01 = No ◯ 88 = Refusal<br>◯ 02 = Yes ◯ 99 = Don't know
6.04	In the last 12 months, have you had symptoms of a sexually transmitted infection including discharge from your penis or sores on or around your penis or anus?	◯ 01 = No[skip to 6.07]<br>◯ 02 = Yes<br>◯ 88 = Refusal [skip to 6.07]<br>◯ 99 = Don't know [skip to 6.07]
	6.05 Was this infection treated by a doctor or other healthcare provider?	◯ 01 = No, no treatment at all ◯ 88 = Refusal<br>◯ 02 = Yes, treated by a healthcare provider ◯ 99 = Don't know<br>◯ 03 = No, self-treated<br>◯ 04 = No, treated by a traditional healer<br>◯ 05 = No, treated by another (specify): ____________
	6.06 When you have had these symptoms from your penis or sores on or around your penis or anus, have you ever avoided seeking care because you are worried others will find out you have sex with men?	◯ 01 = No<br>◯ 02 = Yes<br>◯ 88 = Refusal<br>◯ 99 = Don't know
	Now let's talk about HIV infection	
6.07	Have any of your family members who lived in your household been told by a doctor that they have HIV?	◯ 01 = No [skip to 6.09]<br>◯ 02 = Yes<br>◯ 88 = Refusal [skip to 6.09]<br>◯ 99 = Don't know [skip to 6.09]
6.09	Have ever been tested for HIV infection?	◯ 01 = No [skip to 6.23] ◯ 88 = Refusal [skip to 6.23]<br>◯ 02 = Yes, once ◯ 99 = Don't know [skip to 6.23]<br>◯ 03=Yes, more than once
	6.09a Date when last tested for HIV?	88/8888 = Refusal<br>99/9999 = Don't Know<br>Month __ __ Year __ __ __ __
	6.10 Did you receive the results of your HIV test?	◯ 01 = No [skip to 6.23]<br>◯ 02 = Yes<br>◯ 88 = Refusal [skip to 6.23]<br>◯ 99 = Don't know [skip to 6.23]
	6.11 Have you ever been told by a doctor or healthcare provider that you have HIV after being tested for HIV?	◯ 01 = No [skip to 6.23]<br>◯ 02 = Yes<br>◯ 88 = Refusal [skip to 6.23]<br>◯ 99 = Don't know [skip to 6.23]
	6.12 If you have children, has a doctor or healthcare provider told you that any of your children have HIV?	◯ 01 = No ◯ 88 = Refusal<br>◯ 02 = Yes ◯ 99 = Don't know<br>◯ 03 = Not applicable, no biological children
	6.13 Approximately how many years has it been since you were told by a healthcare provider that you have HIV?	*[Record the number of years]* __ __<br>88 = Refusal<br>99 = Don't know

Study Number:

Visit Date (dd/mm/yyyy) __ __ / __ __ / __ __ __ __

No.	Question	Coded Responses (Shade in the appropriate circles)
6.14	Since the time that you were told that you have HIV, have you told any of these types of healthcare workers your HIV status?	
	6.14a = Doctors	◯ 01 = No<br>◯ 02 = Yes<br>◯ 88 = Refusal<br>◯ 99 = Don't know
	6.14b = Nurses	◯ 01 = No<br>◯ 02 = Yes<br>◯ 88 = Refusal<br>◯ 99 = Don't know
	6.14c = Midwives	◯ 01 = No<br>◯ 02 = Yes<br>◯ 88 = Refusal<br>◯ 99 = Don't know
	6.14d = Counselors	◯ 01 = No<br>◯ 02 = Yes<br>◯ 88 = Refusal<br>◯ 99 = Don't know
	6.14e = Other (specify)	◯ 01 = No<br>◯ 02 = Yes (specify): ____________<br>◯ 88 = Refusal<br>◯ 99 = Don't know
	6.15 To determine when people should begin treatment for HIV, a test called CD4 is done. Have you been tested to learn what your CD4 levels are?	◯ 01 = No, and I am not scheduled to have my CD4 levels monitored [skip to 6.17]<br>◯ 02 = No, but I am scheduled to have my CD4 levels monitored [skip to 6.17]<br>◯ 03 = Yes<br>◯ 88 = Refusal [skip to 6.17]<br>◯ 99 = Don't know [skip to 6.17]
	6.16 Have you received the results of your last CD4 test?	◯ 01 = No<br>◯ 02 = Yes<br>◯ 88 = Refusal<br>◯ 99 = Don't know
	6.17 Have you been told by a physician or healthcare provider that you need to begin treatment for HIV?	◯ 01 = No [skip to 6.21]<br>◯ 02 = Yes<br>◯ 88 = Refusal [skip to 6.21]<br>◯ 99 = Don't know [skip to 6.21]
	6.18 Are you currently being treated for HIV?	◯ 01 = No [skip to 6.21]<br>◯ 02 = Yes<br>◯ 88 = Refusal [skip to 6.21]<br>◯ 99 = Don't know [skip to 6.21]
	6.19 What treatment are you currently receiving?	◯ 01 = Medication from health facility or pharmacy<br>◯ 02 = Traditional herbs<br>◯ 03 = Other (specify): ____________<br>◯ 88 = Refusal<br>◯ 99 = Don't know
	6.20 Has there been a time that you could not obtain all of the medications you need to treat your HIV infection?	◯ 01 = No<br>◯ 02 = Yes<br>◯ 88 = Refusal<br>◯ 99 = Don't know

Study Number: ______________________

Visit Date (dd/mm/yyyy) __ __ / __ __ / __ __ __ __

No.	Question	Coded Responses (Shade in the appropriate circles)
	6.21 Have you told your male sexual partners that you are living with HIV?	○ 01 = No, none<br>○ 02 = Some but not all male sexual partners<br>○ 03 = Yes, all male sexual partners<br>○ 88 = Refusal<br>○ 99 = Don't know
	6.22 Have you told your female sexual partners that you are living with HIV?	○ 01 = No, none<br>○ 02 = Some but not all female sexual partners<br>○ 03 = Yes, all female sexual partners<br>○ 88 = Refusal<br>○ 99 = Don't know
6.23	Do you have access to condoms when you need them?<br>*(By this I mean, are you able to buy them or obtain them for free somewhere?)*	○ 01 = Very difficult access<br>○ 02 = Somewhat difficult access<br>○ 03 = Neither difficult nor easy access<br>○ 04 = Somewhat easy access<br>○ 05 = Very easy access<br>○ 88 = Refusal<br>○ 99 = Don't know
6.24	In general, do you buy condoms or get them for free?	○ 01 = Buy them [skip to 6.29]<br>○ 02 = Get them for free<br>○ 03 = Both<br>○ 04 = Neither<br>○ 88 = Refusal [skip to 6.29]<br>○ 99 = Don't know [skip to 6.29]
	6.26 In the last week, approximately how many condoms did you receive for free?	*[Record the number of condoms]* ☐☐<br>88 = Refusal<br>99 = Don't know
	6.27 Was that quantity that you received for free sufficient?	○ 01 = No<br>○ 02 = Yes<br>○ 88 = Refusal<br>○ 99 = Don't know
6.29	In general, when you use condoms, do you use lubricated condoms?	○ 01 = No<br>○ 02 = Yes<br>○ 88 = Refusal<br>○ 99 = Don't know
6.30	Do you have access to lubricants when you need them?<br>*(By this I mean, are you able to buy them or obtain them for free somewhere?)*<br>*These could include petroleum jelly or Vaseline, body creams/fatty creams, water-based lubricant (ex. KY jelly or lubrica), saliva, or cooking oil.*	○ 01 = Very difficult access<br>○ 02 = Somewhat difficult access<br>○ 03 = Neither difficult nor easy access<br>○ 04 = Somewhat easy access<br>○ 05 = Very easy access<br>○ 88 = Refusal<br>○ 99 = Don't know
	6.31 Have you ever used lubricants during sex?<br>*These could include petroleum jelly or Vaseline, body creams/fatty creams, water-based lubricant (ex. KY jelly or lubrica), saliva, or cooking oil.*	○ 01 = No (skip to 6.36)<br>○ 02 = Yes, without condoms<br>○ 03 = Yes, with condoms<br>○ 04 = Yes, sometimes with and sometimes without condoms<br>○ 88 = Refusal (skip to 6.36)<br>○ 99 = Don't know (skip to 6.36)
6.32	Do you use lubricants with condoms for vaginal or anal sex with women?	○ 01 = No<br>○ 02 = Yes<br>○ 03 = Not applicable - Do not have sex with women<br>○ 88 = Refusal<br>○ 99 = Don't know
	6.33 Which type of lubricant do you most often use for vaginal or anal sex with women?	○ 01 = Petroleum jelly or Vaseline<br>○ 02 = Body creams/fatty creams<br>○ 03 = Water-based lubricant (ex: KY jelly or lubrica)<br>○ 04 = Saliva<br>○ 05 = Cooking oil<br>○ 06 = No lubricant use<br>○ 07 = Other<br>○ 88 = Refusal<br>○ 99 = Don't know<br>(If other, specify): ____________

Study Number: ____________

Visit Date (dd/mm/yyyy) __ __ / __ __ / __ __ __ __

No.	Question	Coded Responses (Shade in the appropriate circles)
6.34	Do you use lubricants with condoms for anal sex with men?	○ 01 = No ○ 88 = Refusal<br>○ 02 = Yes ○ 99 = Don't know
6.35	Which type of lubricant do you most often use for anal sex with men?	○ 01 = Petroleum jelly or Vaseline ○ 06 = No lubricant use<br>○ 02 = Body creams/fatty creams ○ 07 = Other<br>○ 03 = Water-based lubricant (ex: KY jelly or lubrica) ○ 88 = Refusal<br>○ 04 = Saliva ○ 99 = Don't know<br>○ 05 = Cooking oil (If other, specify): ____________
6.36	In the last 12 months, how worried would you say you have been about HIV/AIDS infection?	○ 01 = Very worried ○ 04 = Not at all worried<br>○ 02 = Somewhat worried ○ 88 = Refusal<br>○ 03 = A little worried ○ 99 = Don't know
Now I would like to ask a few questions about drugs or substances that you may have used. Please remember, your responses are private, and we will not share what you have told us with anyone else.		
6.44	Have you ever injected illegal drugs (ex: cocaine, heroin) or legal drugs for recreational purposes?<br><br>*(By recreational purposes, I mean the drug was not prescribed as a medication or for health reasons but you injected it for pleasure, enjoyment, or a high)*	○ 01 = No<br>○ 02 = Yes<br><br>○ 88 = Refusal<br>○ 99 = Don't know
6.46	When using a needle to inject something (for any reason), did you ever share your used needle(s) with another person?	○ 01 = No ○ 88 = Refusal<br>○ 02 = Yes, in the last 12 months ○ 99 = Don't know<br>○ 03 = Yes, but not in the last 12 months
6.47	When using a needle to inject something (for any reason), did you ever use a needle that someone else had already used?	○ 01 = No ○ 88 = Refusal<br>○ 02 = Yes, in the last 12 months ○ 99 = Don't know<br>○ 03 = Yes, but not in the last 12 months
6.49	Have you ever used any drug that you did not inject and that was not prescribed to you for health reasons?<br><br>*(These can include marijuana, banga, glue, powdered cocaine, narcotics, codeine, or other drugs)*	○ 01 = No ○ 88 = Refusal<br>○ 02 = Yes, in the last 12 months ○ 99 = Don't know<br>○ 03 = Yes, but not in the last 12 months
	**6.49a** Do you currently have sex while under the influence of drugs?	○ 01 = No ○ 88 = Refusal<br>○ 02 = Yes, in the last 12 months ○ 99 = Don't know<br>○ 03 = Yes, but not in the last 12 months
	**6.49b** Do you currently smoke cigarettes, cigars, or pipes?	○ 01 = No ○ 88 = Refusal<br>○ 02 = Yes, in the last 12 months ○ 99 = Don't know<br>○ 03 = Yes, but not in the last 12 months
Now I would like to ask a few questions about alcohol you may drink.		
6.50	In the last 30 days, how many days did you drink at least one drink of alcohol?	*[Record the number of days. If 00 (no alcohol use), skip to 6.53]* ☐☐<br><br>88 = Refusal [skip to 6.53]<br>99 = Don't know [skip to 6.53]
6.51	During these days when you did drink alcohol, how many days would you say you drank 5 or more drinks in one sitting?<br><br>One drink is equal to:<br><br>- One 330ml glass, can or bottle of beer<br>- One 50 ml glass or cup of vodka<br>- One 100ml glass of wine<br><br>*[Interviewer check: this response should not be greater than the response for 6.50. If it is, ask both questions again to ensure understanding]*	*[Record the number of days, including 00 if the individual did not drink 5 or more drinks in one sitting on any day in the last 30 days]* ☐☐<br><br>88 = Refusal<br>99 = Don't know

Study Number: ____________ Visit Date (dd/mm/yyyy) __ __ / __ __ / __ __ __ __

No.	Question	Coded Responses (Shade in the appropriate circles)
6.52	Approximately how many drinks containing alcohol do you have on a typical day/night when you are drinking?<br><br>One drink is equal to<br>- One 330ml glass, can or bottle of beer<br>- One 50 ml glass or cup of vodka<br>- One 100ml glass of wine	*[Record the number of drinks. E.g. 04 for 4 drinks. If a respondent had consumed three bottles of beer each 330ml and one 100ml glass of wine, mark as 04.]* ☐☐<br><br>88 = Refusal<br>99 = Don't know
	6.52a Do you currently have sex while under the influence of alcohol?	○ 01 = No<br>○ 02 = Yes, in the last 12 months<br>○ 03 = Yes, but not in the last 12 months<br>○ 88 = Refusal<br>○ 99 = Don't know
Now I would like to ask a few questions about sexual practices and HIV.		
6.53	What type of sex puts you most at risk for HIV infection?	○ 01 = Vaginal sex<br>○ 02 = Anal sex<br>○ 03 = Oral sex<br>○ 04 = All carry equal risk<br>○ 88 = Refusal<br>○ 99 = Don't know
6.54	Which type of anal sex position puts you most at risk for HIV infection?	○ 01 = Insertive (top)<br>○ 02 = Receptive (bottom)<br>○ 03 = Insertive and receptive anal sex carry equal risk<br>○ 88 = Refusal<br>○ 99 = Don't know
6.55	Which is the safest lubricant to use during vaginal sex with a woman with latex condoms?	○ 01 = Petroleum jelly or Vaseline<br>○ 02 = Body creams/fatty creams<br>○ 03 = Water-based lubricant (ex: Durex, Vendome, Manex)<br>○ 04 = Saliva<br>○ 05 = Cooking oil<br>○ 06 = No lubricant use<br>○ 07 = KY/lubrica<br>○ 08 = Other<br>○ 88 = Refusal<br>○ 99 = Don't know<br>(If other, specify): ____________
6.56	Which is the safest lubricant to use during anal sex with latex condoms?	○ 01 = Petroleum jelly or Vaseline<br>○ 02 = Body creams/fatty creams<br>○ 03 = Water-based lubricant (ex: Durex, Vendome, Manex)<br>○ 04 = Saliva<br>○ 05 = Cooking oil<br>○ 06 = No lubricant use<br>○ 07 = KY/lubrica<br>○ 08 = Other<br>○ 88 = Refusal<br>○ 99 = Don't know<br>(If other, specify): ____________
6.57	Can you get HIV from using a needle to inject a drug or substance after someone else has used the needle?	○ 01 = No<br>○ 02 = Yes<br><br>○ 88 = Refusal<br>○ 99 = Don't know

End of questionnaire

"Thank you for taking the time to sit with me. I know we have gone through many questions and I really appreciate your time and openness. We are finished with the long part of the study. Do you feel ready to proceed with clinical care?"

Escort participant to coupon manager
