# Supplementary material for: HIV status disclosure by Nigerian men who have sex with men and transgender women living with HIV: a cross-sectional analysis at enrollment into an observational cohort
Source: BMC Public Health. 2020 Aug 26;20:1282. doi: 10.1186/s12889-020-09315-y (PMC7448976; doi:10.1186/s12889-020-09315-y)
Supplement: Supplementary file 2 — Additional file 2: Supplementary File 2. TRUST/RV368 Questionnaire (Visit 1). Participant demographics, perceptions and experiences of stigma, and sexual behaviors were evaluated using questionnaires developed specifically for this study that were administered by a trained interviewer over the first two study visits, approximately two weeks apart. The second set of questions (Visit 1) covered sexual network risks; composition and influence of social networks; condom negotiation; social capital; and exposure to health information. [file 12889_2020_9315_MOESM2_ESM.pdf]

Visit Date (dd/mm/yyyy) __ __ / __ __ / __ __ __ __	Interviewer ID __ __	Location: ○ CHC ○ 68 NARHY
Study Number:	RDS Coupon Number used to recruit participant __ __ __ __ __ __ __ __	

MODULE 7 - Risk in MSM Sexual Network

The next set of questions will ask you more details about the men with whom you have had anal or oral sex with in the past year.

No.	Question	Coded Responses (Shade in the appropriate circles)
7.01	In all with how many different men did you have anal or oral sex with in the past 12 months?	*[Record the number of men. If 000 skip to 8.01]*<br>888 = Refusal [skip to 8.01]<br>999 = Don't know [skip to 8.01]<br>__ __ __

Can you tell me the initials or use a pseudonym for the five men you most recently had anal or oral sex with in the past year, starting with the man with whom you most recently had anal or oral sex. A pseudonym is a fake name that you can use in place of their real name. If you use a pseudonym, use one that will allow you to remember the person that you are using it for. All the questions are regarding the period that you maintained a relationship with each partner, unless specified in contrary.

ENTER INITIALS OR PSEUDONYM OF FIVE MOST RESENT SEXUAL PARTNERS

1. __________ 2. __________ 3. __________

4. __________ 5. __________

	FIVE MOST RESENT SEXUAL PARTNERS				
	1.	2.	3.	4.	5.
7.02 Record [NAME] age in years<br>88 = Refusal<br>99 = Don't Know	__ __	__ __	__ __	__ __	__ __
7.021 How old is [NAME] relative to you?<br>01 = Older<br>02 = Same age<br>03 = Younger<br>88 = Refusal<br>99 = Don't know	○ 01<br>○ 02<br>○ 03<br>○ 88<br>○ 99	○ 01<br>○ 02<br>○ 03<br>○ 88<br>○ 99	○ 01<br>○ 02<br>○ 03<br>○ 88<br>○ 99	○ 01<br>○ 02<br>○ 03<br>○ 88<br>○ 99	○ 01<br>○ 02<br>○ 03<br>○ 88<br>○ 99
7.03 [NAME] level of education:<br>00 = Never been to school<br>01 = Quranic<br>02 = Primary<br>03 = Junior Secondary<br>04 = Senior Secondary<br>05 = Higher then Senior Secondary<br>88 = Refusal<br>99 = Don't Know	○ 00<br>○ 01<br>○ 02<br>○ 03<br>○ 04<br>○ 05<br>○ 88<br>○ 99	○ 00<br>○ 01<br>○ 02<br>○ 03<br>○ 04<br>○ 05<br>○ 88<br>○ 99	○ 00<br>○ 01<br>○ 02<br>○ 03<br>○ 04<br>○ 05<br>○ 88<br>○ 99	○ 00<br>○ 01<br>○ 02<br>○ 03<br>○ 04<br>○ 05<br>○ 88<br>○ 99	○ 00<br>○ 01<br>○ 02<br>○ 03<br>○ 04<br>○ 05<br>○ 88<br>○ 99
7.031 What is [NAME]'s religion?<br>01 = Protestant/other Christian<br>02 = Muslim<br>03 = No religion<br>04 = Other<br>88 = Refusal<br>99 = Don't know	○ 01<br>○ 02<br>○ 03<br>○ 04<br>○ 88<br>○ 99	○ 01<br>○ 02<br>○ 03<br>○ 04<br>○ 88<br>○ 99	○ 01<br>○ 02<br>○ 03<br>○ 04<br>○ 88<br>○ 99	○ 01<br>○ 02<br>○ 03<br>○ 04<br>○ 88<br>○ 99	○ 01<br>○ 02<br>○ 03<br>○ 04<br>○ 88<br>○ 99

Study Number:

Visit Date (dd/mm/yyyy) __ / __ / ____

	FIVE MOST RESENT SEXUAL PARTNERS				
	1.	2.	3.	4.	5.
7.032 What is [NAME]'s occupation?<br>01 = Not working<br>02 = Pupil/student<br>03 = Professional<br>04 = Self employed<br>05 = Entertainment/service<br>06 = Driver/laborer<br>07 = Military/police<br>08 = Other<br>88 = Refusal<br>99 = Don't know	○ 01<br>○ 02<br>○ 03<br>○ 04<br>○ 05<br>○ 06<br>○ 07<br>○ 08<br>○ 88<br>○ 99	○ 01<br>○ 02<br>○ 03<br>○ 04<br>○ 05<br>○ 06<br>○ 07<br>○ 08<br>○ 88<br>○ 99	○ 01<br>○ 02<br>○ 03<br>○ 04<br>○ 05<br>○ 06<br>○ 07<br>○ 08<br>○ 88<br>○ 99	○ 01<br>○ 02<br>○ 03<br>○ 04<br>○ 05<br>○ 06<br>○ 07<br>○ 08<br>○ 88<br>○ 99	○ 01<br>○ 02<br>○ 03<br>○ 04<br>○ 05<br>○ 06<br>○ 07<br>○ 08<br>○ 88<br>○ 99
7.04 [NAME] marital status during the relationship period?<br>01 = Married to a woman<br>02 = Cohabitating<br>03 = Separated or divorced<br>04 = Widowed<br>05 = Singe or Married<br>88 = Refusal<br>99 = Don't know	○ 01<br>○ 02<br>○ 03<br>○ 04<br>○ 05<br>○ 88<br>○ 99	○ 01<br>○ 02<br>○ 03<br>○ 04<br>○ 05<br>○ 88<br>○ 99	○ 01<br>○ 02<br>○ 03<br>○ 04<br>○ 05<br>○ 88<br>○ 99	○ 01<br>○ 02<br>○ 03<br>○ 04<br>○ 05<br>○ 88<br>○ 99	○ 01<br>○ 02<br>○ 03<br>○ 04<br>○ 05<br>○ 88<br>○ 99
7.05 How would you classify [NAME] socio-economic level, compared to yours?<br>01 = Lower<br>02 = The same<br>03 = Higher<br>88 = Refusal<br>99 = Don't Know	○ 01<br>○ 02<br>○ 03<br>○ 88<br>○ 99	○ 01<br>○ 02<br>○ 03<br>○ 88<br>○ 99	○ 01<br>○ 02<br>○ 03<br>○ 88<br>○ 99	○ 01<br>○ 02<br>○ 03<br>○ 88<br>○ 99	○ 01<br>○ 02<br>○ 03<br>○ 88<br>○ 99
7.06 Rate the strength of friendship with [NAME] from 0 to 10,<br>[0 being 'acquaintance' and 10 being 'best friend']<br>88 = Refusal<br>99 = Don't Know	☐☐	☐☐	☐☐	☐☐	☐☐
7.061 How much do you trust [NAME]?<br>00 = Do not trust<br>01 = Neutral<br>02 = A little trust<br>03 = A lot of trust<br>88 = Refusal<br>99 = Don't know	○ 00<br>○ 01<br>○ 02<br>○ 03<br>○ 88<br>○ 99	○ 00<br>○ 01<br>○ 02<br>○ 03<br>○ 88<br>○ 99	○ 00<br>○ 01<br>○ 02<br>○ 03<br>○ 88<br>○ 99	○ 00<br>○ 01<br>○ 02<br>○ 03<br>○ 88<br>○ 99	○ 00<br>○ 01<br>○ 02<br>○ 03<br>○ 88<br>○ 99
7.07 [NAME] is what type of sexual partner:<br>01 = Regular<br>02 = Casual (By casual partner, this means a man that you have sex with, but you don't feel committed to)<br>88 = Refusal<br>99 = Don't know	○ 01<br>○ 02<br>○ 88<br>○ 99	○ 01<br>○ 02<br>○ 88<br>○ 99	○ 01<br>○ 02<br>○ 88<br>○ 99	○ 01<br>○ 02<br>○ 88<br>○ 99	○ 01<br>○ 02<br>○ 88<br>○ 99
7.071 What month and year did your relationship with [NAME] begin ?<br>88 = Refusal<br>99 = Don't know	☐☐ (month)<br>☐☐ (year)	☐☐ (month)<br>☐☐ (year)	☐☐ (month)<br>☐☐ (year)	☐☐ (month)<br>☐☐ (year)	☐☐ (month)<br>☐☐ (year)
7.072 What month and year did your relationship with [NAME] end (MMYY)?<br>00 = Relationship still ongoing<br>88 = Refusal<br>99 = Don't know	☐☐ (month)<br>☐☐ (year)	☐☐ (month)<br>☐☐ (year)	☐☐ (month)<br>☐☐ (year)	☐☐ (month)<br>☐☐ (year)	☐☐ (month)<br>☐☐ (year)
7.073 Did you meet [NAME] through the internet?<br>01 = No<br>02 = Yes<br>88 = Refusal<br>99 = Don't know	○ 01<br>○ 02<br>○ 88<br>○ 99	○ 01<br>○ 02<br>○ 88<br>○ 99	○ 01<br>○ 02<br>○ 88<br>○ 99	○ 01<br>○ 02<br>○ 88<br>○ 99	○ 01<br>○ 02<br>○ 88<br>○ 99

TRUST QUESTIONNAIRE

Module 7 - Risk in MSM Sexual Network

Study Number: ____________

Visit Date (dd/mm/yyyy) __ __ / __ __ / __ __ __ __

	FIVE MOST RESENT SEXUAL PARTNERS				
	1.	2.	3.	4.	5.
7.08 How frequently did you have sex with [NAME]?<br>01 = Almost every day<br>02 = A few times each week<br>03 = A few times each month<br>04 = Once a month or fewer times<br>77 = I had sex only once or twice with this partner<br>88 = Refusal<br>99 = Don't Know	○ 01<br>○ 02<br>○ 03<br>○ 04<br>○ 77<br>○ 88<br>○ 99	○ 01<br>○ 02<br>○ 03<br>○ 04<br>○ 77<br>○ 88<br>○ 99	○ 01<br>○ 02<br>○ 03<br>○ 04<br>○ 77<br>○ 88<br>○ 99	○ 01<br>○ 02<br>○ 03<br>○ 04<br>○ 77<br>○ 88<br>○ 99	○ 01<br>○ 02<br>○ 03<br>○ 04<br>○ 77<br>○ 88<br>○ 99
7.081 Does [NAME] encourage you to use condoms?<br>01 = No<br>02 = Yes<br>88 = Refusal<br>99 = Don't know	○ 01<br>○ 02<br>○ 88<br>○ 99	○ 01<br>○ 02<br>○ 88<br>○ 99	○ 01<br>○ 02<br>○ 88<br>○ 99	○ 01<br>○ 02<br>○ 88<br>○ 99	○ 01<br>○ 02<br>○ 88<br>○ 99
7.082 If you are HIV negative, does [NAME] encourage you to get tested for HIV?<br>01 = No<br>02 = Yes<br>88 = Refusal<br>99 = Don't know	○ 01<br>○ 02<br>○ 88<br>○ 99	○ 01<br>○ 02<br>○ 88<br>○ 99	○ 01<br>○ 02<br>○ 88<br>○ 99	○ 01<br>○ 02<br>○ 88<br>○ 99	○ 01<br>○ 02<br>○ 88<br>○ 99
7.09 How would you define [NAME] sexual orientation?<br>01 = Homosexual (gay)<br>02 = Bisexual<br>03 = Heterosexual<br>04 = Transgender<br>88 = Refusal<br>99 = Don't Know	○ 01<br>○ 02<br>○ 03<br>○ 04<br>○ 88<br>○ 99	○ 01<br>○ 02<br>○ 03<br>○ 04<br>○ 88<br>○ 99	○ 01<br>○ 02<br>○ 03<br>○ 04<br>○ 88<br>○ 99	○ 01<br>○ 02<br>○ 03<br>○ 04<br>○ 88<br>○ 99	○ 01<br>○ 02<br>○ 03<br>○ 04<br>○ 88<br>○ 99
7.091 What does [NAME] consider to be his gender?<br>01 = Man<br>02 = Woman<br>03 = Other (if other, specify below)<br>04 = Both man and woman<br>88 = Refusal<br>99 = Don't Know	○ 01<br>○ 02<br>○ 03<br>○ 04<br>○ 88<br>○ 99	○ 01<br>○ 02<br>○ 03<br>○ 04<br>○ 88<br>○ 99	○ 01<br>○ 02<br>○ 03<br>○ 04<br>○ 88<br>○ 99	○ 01<br>○ 02<br>○ 03<br>○ 04<br>○ 88<br>○ 99	○ 01<br>○ 02<br>○ 03<br>○ 04<br>○ 88<br>○ 99

If other gender (03) selected above, please specify: Other 1: ____________ Other 2: ____________

Other 3: ____________ Other 4: ____________ Other 5: ____________

	1.	2.	3.	4.	5.
7.10 How would you rate [NAME] chance of acquiring the AIDS virus from 0 (impossible) to 10 (I think he/she has AIDS)?<br>88 = Refusal<br>99 = Don't know	☐☐	☐☐	☐☐	☐☐	☐☐
7.11 As far as you know, is [NAME] living with HIV?<br>01 = No<br>02 = Yes<br>88 = Refusal<br>99 = Don't Know	○ 01<br>○ 02<br>○ 88<br>○ 99	○ 01<br>○ 02<br>○ 88<br>○ 99	○ 01<br>○ 02<br>○ 88<br>○ 99	○ 01<br>○ 02<br>○ 88<br>○ 99	○ 01<br>○ 02<br>○ 88<br>○ 99
7.12 As far as you know, does [NAME] have any kind of sexually transmitted infection such as syphilis, gonorrhea or genital wart?<br>01 = No<br>02 = Yes<br>88 = Refusal<br>99 = Don't Know	○ 01<br>○ 02<br>○ 88<br>○ 99	○ 01<br>○ 02<br>○ 88<br>○ 99	○ 01<br>○ 02<br>○ 88<br>○ 99	○ 01<br>○ 02<br>○ 88<br>○ 99	○ 01<br>○ 02<br>○ 88<br>○ 99
7.13 If you had oral sex with [NAME], how often was a condom used?<br>01 = Never<br>02 = Almost never<br>03 = About half the time<br>04 = Almost always<br>05 = Always<br>77 = Did not have oral sex with this partner<br>88 = Refusal<br>99 = Don't Know	○ 01<br>○ 02<br>○ 03<br>○ 04<br>○ 05<br>○ 77<br>○ 88<br>○ 99	○ 01<br>○ 02<br>○ 03<br>○ 04<br>○ 05<br>○ 77<br>○ 88<br>○ 99	○ 01<br>○ 02<br>○ 03<br>○ 04<br>○ 05<br>○ 77<br>○ 88<br>○ 99	○ 01<br>○ 02<br>○ 03<br>○ 04<br>○ 05<br>○ 77<br>○ 88<br>○ 99	○ 01<br>○ 02<br>○ 03<br>○ 04<br>○ 05<br>○ 77<br>○ 88<br>○ 99

Study Number: ______________ Visit Date (dd/mm/yyyy) __ __ / __ __ / __ __ __ __

	FIVE MOST RESENT SEXUAL PARTNERS				
	1.	2.	3.	4.	5.
7.14 When you had receptive anal sex with [NAME] (your partner's penis introduced in your anus), how often was a condom used?<br>01 = Never<br>02 = Almost never<br>03 = About half the time<br>04 = Almost always<br>05 = Always<br>77 = Did not have receptive anal sex with this partner<br>88 = Refusal<br>99 = Don't know	○ 01<br>○ 02<br>○ 03<br>○ 04<br>○ 05<br>○ 77<br>○ 88<br>○ 99	○ 01<br>○ 02<br>○ 03<br>○ 04<br>○ 05<br>○ 77<br>○ 88<br>○ 99	○ 01<br>○ 02<br>○ 03<br>○ 04<br>○ 05<br>○ 77<br>○ 88<br>○ 99	○ 01<br>○ 02<br>○ 03<br>○ 04<br>○ 05<br>○ 77<br>○ 88<br>○ 99	○ 01<br>○ 02<br>○ 03<br>○ 04<br>○ 05<br>○ 77<br>○ 88<br>○ 99
7.15 When you had insertive anal sex with [NAME] (your penis introduced in your partner's anus), how often was a condom used?<br>01 = Never<br>02 = Almost never<br>03 = About half the time<br>04 = Almost always<br>05 = Always<br>77 = Did not have insertive anal sex with this partner<br>88 = Refusal<br>99 = Don't Know	○ 01<br>○ 02<br>○ 03<br>○ 04<br>○ 05<br>○ 77<br>○ 88<br>○ 99	○ 01<br>○ 02<br>○ 03<br>○ 04<br>○ 05<br>○ 77<br>○ 88<br>○ 99	○ 01<br>○ 02<br>○ 03<br>○ 04<br>○ 05<br>○ 77<br>○ 88<br>○ 99	○ 01<br>○ 02<br>○ 03<br>○ 04<br>○ 05<br>○ 77<br>○ 88<br>○ 99	○ 01<br>○ 02<br>○ 03<br>○ 04<br>○ 05<br>○ 77<br>○ 88<br>○ 99
7.17 As far as you know, did [NAME] have more than one sexual regular partner at the same time (for example, you and a wife or a regular lover)?<br>01 = No<br>02 = Yes<br>88 = Refusal<br>99 = Don't Know	○ 01<br>○ 02<br>○ 88<br>○ 99	○ 01<br>○ 02<br>○ 88<br>○ 99	○ 01<br>○ 02<br>○ 88<br>○ 99	○ 01<br>○ 02<br>○ 88<br>○ 99	○ 01<br>○ 02<br>○ 88<br>○ 99
7.201 Did you have sex the first time you met [NAME]?<br>01 = No<br>02 = Yes<br>88 = Refusal<br>99 = Don't Know	○ 01<br>○ 02<br>○ 88<br>○ 99	○ 01<br>○ 02<br>○ 88<br>○ 99	○ 01<br>○ 02<br>○ 88<br>○ 99	○ 01<br>○ 02<br>○ 88<br>○ 99	○ 01<br>○ 02<br>○ 88<br>○ 99
7.211 Did you pay money, favors or drugs to [NAME] to have sex?<br>01 = No<br>02 = Yes<br>88 = Refusal<br>99 = Don't Know	○ 01<br>○ 02<br>○ 88<br>○ 99	○ 01<br>○ 02<br>○ 88<br>○ 99	○ 01<br>○ 02<br>○ 88<br>○ 99	○ 01<br>○ 02<br>○ 88<br>○ 99	○ 01<br>○ 02<br>○ 88<br>○ 99
7.221 Did you receive money, favors or drugs from {NAME] in exchange for sex?<br>01 = No<br>02 = Yes<br>88 = Refusal<br>99 = Don't Know	○ 01<br>○ 02<br>○ 88<br>○ 99	○ 01<br>○ 02<br>○ 88<br>○ 99	○ 01<br>○ 02<br>○ 88<br>○ 99	○ 01<br>○ 02<br>○ 88<br>○ 99	○ 01<br>○ 02<br>○ 88<br>○ 99
7.231 Did you have sex with [NAME] while under alcohol or drug influence?<br>01 = No<br>02 = Yes<br>88 = Refusal<br>99 = Don't Know	○ 01<br>○ 02<br>○ 88<br>○ 99	○ 01<br>○ 02<br>○ 88<br>○ 99	○ 01<br>○ 02<br>○ 88<br>○ 99	○ 01<br>○ 02<br>○ 88<br>○ 99	○ 01<br>○ 02<br>○ 88<br>○ 99
7.232 Has [NAME] had sex with [Person 2] in the past 12 months?<br>01 = No<br>02 = Yes<br>88 = Refusal<br>99 = Don't know	○ 01<br>○ 02<br>○ 88<br>○ 99				
7.233 Has [NAME] had sex with [Person 3] in the past 12 months?<br>01 = No<br>02 = Yes<br>88 = Refusal<br>99 = Don't know	○ 01<br>○ 02<br>○ 88<br>○ 99	○ 01<br>○ 02<br>○ 88<br>○ 99			
7.234 Has [NAME] had sex with [Person 4] in the past 12 months?<br>01 = No<br>02 = Yes<br>88 = Refusal<br>99 = Don't know	○ 01<br>○ 02<br>○ 88<br>○ 99	○ 01<br>○ 02<br>○ 88<br>○ 99	○ 01<br>○ 02<br>○ 88<br>○ 99		

Study Number: ____________

Visit Date (dd/mm/yyyy) __ __ / __ __ / __ __ __ __

	FIVE MOST RESENT SEXUAL PARTNERS				
	1.	2.	3.	4.	5.
7.235 Has [NAME] had sex with [Person 5] in the past 12 months?<br>01 = No<br>02 = Yes<br>88 = Refusal<br>99 = Don't know	○ 01<br>○ 02<br>○ 88<br>○ 99	○ 01<br>○ 02<br>○ 88<br>○ 99	○ 01<br>○ 02<br>○ 88<br>○ 99	○ 01<br>○ 02<br>○ 88<br>○ 99	
7.27 From 0 (not sure at all) to 10 (100% sure), how sure are you about the information you have just given on [NAME]?<br>88 = Refusal<br>99 = Don't know	☐☐	☐☐	☐☐	☐☐	☐☐

MODULE 8 - Composition and Influence of MSM Social Network

8.01 Now, I would like you to think of all of the men that you know who have sex with other men? Can you tell me the initials or use a pseudonym for the five men you know who have sex with other men and with whom you would be most likely to discuss personal matters of importance to you. A pseudonym is a fake name that you can use in place of their real name. If you use a pseudonym, use one that will allow you to remember the person that you are using it for.

ENTER INITIALS OR PSEUDONYM OF FIVE CLOSEST MSM FRIENDS IN THE SPACE PROVIDED BELOW

1. ☐☐☐☐☐☐☐☐☐☐
2. ☐☐☐☐☐☐☐☐☐☐
3. ☐☐☐☐☐☐☐☐☐☐
4. ☐☐☐☐☐☐☐☐☐☐
5. ☐☐☐☐☐☐☐☐☐☐

	FIVE MOST RESENT SEXUAL PARTNERS				
	1.	2.	3.	4.	5.
8.02 How frequently do you discuss HIV with [EACH PERSON]<br>01 = At least once a week<br>02 = At least once a month<br>03 = Less than once a month<br>04 = Never<br>88 = Refusal<br>99 = Don't know this person?	○ 01<br>○ 02<br>○ 03<br>○ 04<br>○ 88<br>○ 99	○ 01<br>○ 02<br>○ 03<br>○ 04<br>○ 88<br>○ 99	○ 01<br>○ 02<br>○ 03<br>○ 04<br>○ 88<br>○ 99	○ 01<br>○ 02<br>○ 03<br>○ 04<br>○ 88<br>○ 99	○ 01<br>○ 02<br>○ 03<br>○ 04<br>○ 88<br>○ 99
8.04 Has [NAME] ever encouraged you to make sure a condom is used when you have sex with another man?<br>01 = No<br>02 = Yes<br>88 = Refused<br>99 = Don't know	○ 01<br>○ 02<br>○ 88<br>○ 99	○ 01<br>○ 02<br>○ 88<br>○ 99	○ 01<br>○ 02<br>○ 88<br>○ 99	○ 01<br>○ 02<br>○ 88<br>○ 99	○ 01<br>○ 02<br>○ 88<br>○ 99
8.05 Does [NAME] know (Person 2)?<br>01 = No<br>02 = Yes<br>88 = Refusal<br>99 = Don't know	○ 01<br>○ 02<br>○ 88<br>○ 99				
8.06 Does [NAME] know (Person 3)?<br>01 = No<br>02 = Yes<br>88 = Refusal<br>99 = Don't know	○ 01<br>○ 02<br>○ 88<br>○ 99	○ 01<br>○ 02<br>○ 88<br>○ 99			
8.07 Does [NAME] know (Person 4)?<br>01 = No<br>02 = Yes<br>88 = Refusal<br>99 = Don't know	○ 01<br>○ 02<br>○ 88<br>○ 99	○ 01<br>○ 02<br>○ 88<br>○ 99	○ 01<br>○ 02<br>○ 88<br>○ 99		
8.08 Does [NAME] know (Person 5)?<br>01 = No<br>02 = Yes<br>88 = Refusal<br>99 = Don't know	○ 01<br>○ 02<br>○ 88<br>○ 99	○ 01<br>○ 02<br>○ 88<br>○ 99	○ 01<br>○ 02<br>○ 88<br>○ 99	○ 01<br>○ 02<br>○ 88<br>○ 99	

TRUST QUESTIONNAIRE

Module 9 - Condom Negotiation

Study Number: ______ Visit Date (dd/mm/yyyy) __ __ / __ __ / __ __ __ __

"I'm going to ask you several questions about using condoms and how difficult you may find it to negotiate condom use in different circumstances. For each situation, you can tell me if you find it very difficult, somewhat difficult, neither difficult nor easy, somewhat easy or very easy."

No.	Question	Coded Responses (Shade in the appropriate circles)	
9.07	How difficult or easy is it for you to insist on condom use with a male sexual partner with whom you haven't always used condoms in the past?	◯ 01 = Very difficult<br>◯ 02 = Somewhat difficult<br>◯ 03 = Neither difficult nor easy<br>◯ 04 = Somewhat easy	◯ 05 = Very easy<br>◯ 77 = Not applicable<br>◯ 88 = Refusal<br>◯ 99 = Don't know
9.10	How difficult or easy is it for you to insist on condom use with a male sexual partner that provides you with regular, economic support?	◯ 01 = Very difficult<br>◯ 02 = Somewhat difficult<br>◯ 03 = Neither difficult nor easy<br>◯ 04 = Somewhat easy	◯ 05 = Very easy<br>◯ 77 = Not applicable<br>◯ 88 = Refusal<br>◯ 99 = Don't know
9.12	How difficult or easy is it for you to negotiate using a condom during oral sex with your male sexual partner?	◯ 01 = Very difficult<br>◯ 02 = Somewhat difficult<br>◯ 03 = Neither difficult nor easy<br>◯ 04 = Somewhat easy	◯ 05 = Very easy<br>◯ 77 = Not applicable<br>◯ 88 = Refusal<br>◯ 99 = Don't know

Module 10 - Social Capital

"Now, I would like to ask you some questions about your social life with other MSM in your group of friends. I'm going to read some phrases and you can tell me if you strongly agree, mostly agree, mostly disagree, or strongly disagree."

No.	Question	Coded Responses (Shade in the appropriate circles)	
10.01	You can count on other MSM in your group of friends if you need to borrow money.	◯ 00 = Strongly disagree<br>◯ 01 = Disagree<br>◯ 02 = Agree	◯ 03 = Strongly agree<br>◯ 88 = Refusal<br>◯ 99 = Don't know
10.02	You can count on other MSM in your group of friends to accompany you to the doctor or hospital	◯ 00 = Strongly disagree<br>◯ 01 = Disagree<br>◯ 02 = Agree	◯ 03 = Strongly agree<br>◯ 88 = Refusal<br>◯ 99 = Don't know
10.03	You can count on other MSM in your group of friends if you need to talk about your problems.	◯ 00 = Strongly disagree<br>◯ 01 = Disagree<br>◯ 02 = Agree	◯ 03 = Strongly agree<br>◯ 88 = Refusal<br>◯ 99 = Don't know
10.04	In general, MSM in your group of friends only worry about themselves.	◯ 00 = Strongly disagree<br>◯ 01 = Disagree<br>◯ 02 = Agree	◯ 03 = Strongly agree<br>◯ 88 = Refusal<br>◯ 99 = Don't know
10.05	You can count on other MSM in your group of friends if you need somewhere to stay.	◯ 00 = Strongly disagree<br>◯ 01 = Disagree<br>◯ 02 = Agree	◯ 03 = Strongly agree<br>◯ 88 = Refusal<br>◯ 99 = Don't know
10.06	You can count on other MSM in your group of friends to help deal with a violent or difficult situation.	◯ 00 = Strongly disagree<br>◯ 01 = Disagree<br>◯ 02 = Agree	◯ 03 = Strongly agree<br>◯ 88 = Refusal<br>◯ 99 = Don't know
10.07	You can count on other MSM in your group of friends to help you find other MSM.	◯ 00 = Strongly disagree<br>◯ 01 = Disagree<br>◯ 02 = Agree	◯ 03 = Strongly agree<br>◯ 88 = Refusal<br>◯ 99 = Don't know
10.08	You can count on other MSM in your group of friends to support the use of condoms.	◯ 00 = Strongly disagree<br>◯ 01 = Disagree<br>◯ 02 = Agree	◯ 03 = Strongly agree<br>◯ 88 = Refusal<br>◯ 99 = Don't know
10.09	The group of friends with whom you socialize with is a mix of straight people and MSM.	◯ 00 = Strongly disagree<br>◯ 01 = Disagree<br>◯ 02 = Agree	◯ 03 = Strongly agree<br>◯ 88 = Refusal<br>◯ 99 = Don't know

MODULE 10 - Social Capital

Study Number: ____________

Visit Date (dd/mm/yyyy) __ __ / __ __ / __ __ __ __

No.	Question	Coded Responses (Shade in the appropriate circles)
10.10	In general the MSM you socialize with are always arguing amongst each other.	○ 00 = Strongly disagree<br>○ 01 = Disagree<br>○ 02 = Agree<br>○ 03 = Strongly agree<br>○ 88 = Refusal<br>○ 99 = Don't know
10.11	You can trust the majority of the MSM you know.	○ 00 = Strongly disagree<br>○ 01 = Disagree<br>○ 02 = Agree<br>○ 03 = Strongly agree<br>○ 88 = Refusal<br>○ 99 = Don't know
10.13	In the past 12 months, how often have you participated in a meeting, march, rally, or gathering to promote the rights of MSM?	○ 00 = Never<br>○ 01 = Once<br>○ 02 = A couple of times<br>○ 03 = Frequently<br>○ 88 = Refusal<br>○ 99 = Don't know
10.14	In the past 12 months, how often have you gotten together with other men who have sex with men to speak with government officials or political leaders to address a problem or common issue facing MSM?	○ 00 = Never<br>○ 01 = Once<br>○ 02 = A couple of times<br>○ 03 = Frequently<br>○ 88 = Refusal<br>○ 99 = Don't know
10.15	In the past 12 months, how often have you joined together with other MSM to address a common problem facing MSM?	○ 00 = Never<br>○ 01 = Once<br>○ 02 = A couple of times<br>○ 03 = Frequently<br>○ 88 = Refusal<br>○ 99 = Don't know
10.16	In the past 12 months, how often have you participated in an HIV prevention organization or MSM rights group?	○ 00 = Never<br>○ 01 = Once<br>○ 02 = A couple of times<br>○ 03 = Frequently<br>○ 88 = Refusal<br>○ 99 = Don't know
10.22	In the past 12 months, how often have you gone into a gay or bisexual chat room on the internet?<br>Was it:	○ 01 = Did not go<br>○ 02 = Once a month or less<br>○ 03 = About once a week<br>○ 04 = Several times a week<br>○ 05 = About once a day<br>○ 06 = Several times a day<br>○ 88 = Refusal<br>○ 99 = Don't know
10.23	In the past 12 months, how often have you used the Internet to look for male sexual partners?<br>Was it:	○ 01 = Did not use [skip to module 11]<br>○ 02 = Once a month or less<br>○ 03 = About once a week<br>○ 04 = Several times a week<br>○ 05 = About once a day<br>○ 06 = Several times a day<br>○ 88 = Refusal [skip to module 11]<br>○ 99 = Don't know [skip to module 11]
	10.24 Which social networking sites did you mainly use?<br><br>(Select all that apply by shading in the circles provided. If other, refusal or don't know, then fill in the box provided)	○ Facebook<br>○ Gay.com<br>○ Blackberry messenger<br>○ Twitter<br>○ Gaydar.co.uk<br>○ Manjam.com<br>○ Badoo.com<br>○ Whatsapp (Mobile app)<br>○ 2GO<br>○ bdclive.com<br>○ Other<br>○ Refusal<br>○ Don't know<br><br>(If other, specify): ____________
	10.25 What devices did you mainly use?<br><br>(Select all that apply by shading in the circles provided. If other, refusal or don't know, then fill in the box provided)	○ Desktop/Laptop/tablet<br>○ Smartphone/Phablet<br>○ Feature phone<br>○ Other<br>○ Refusal<br>○ Don't know<br><br>(If other, specify): ____________

Study Number: ______________ Visit Date (dd/mm/yyyy) __ __ / __ __ / __ __ __ __

"In this next section, I will be asking you some questions so that I can understand how much health information you may have been exposed to. This is the last section and it will be very short"

No.	Question	Coded Responses (Shade in the appropriate circles)
11.01	In the last 12 months, have you received information on prevention of HIV infection from sex between men and women?	○ 01 = No [skip to 11.03]<br>○ 02 = Yes<br>○ 88 = Refusal [skip to 11.03]<br>○ 99 = Don't know [skip to 11.03]
	11.02 Where did you receive this information?	○ 01 = Health Facility<br>○ 02 = Peer Educator/workshop<br>○ 03 = School<br>○ 04 = Internet<br>○ 05 = Friends<br>○ 06 = Media<br>○ 88 = Refusal<br>○ 99 = Don't know
11.03	In the last 12 months, have you received information on prevention of HIV infection from sex between men?	○ 01 = No [skip to 11.05]<br>○ 02 = Yes<br>○ 88 = Refusal [skip to 11.05]<br>○ 99 = Don't know [skip to 11.05]
	11.04 Where did you get this information?	○ 01 = Health Facility<br>○ 02 = Peer Educator/workshop<br>○ 03 = School<br>○ 04 = Internet<br>○ 05 = Friends<br>○ 06 = Media<br>○ 88 = Refusal<br>○ 99 = Don't know
11.05	In the last 12 months, how worried would you say you have been about HIV/AIDS?	○ 01 = Not at all worried<br>○ 02 = Not very worried<br>○ 03 = Somewhat worried<br>○ 04 = Very worried<br>○ 88 = Refusal<br>○ 99 = Don't know
11.06	In the last 12 months, have you participated in any talks or meetings related to HIV/AIDS?	○ 01 = No [skip to 11.08]<br>○ 02 = Yes<br>○ 88 = Refusal [skip to 11.08]<br>○ 99 = Don't know [skip to 11.08]
	11.07 Who hosted these talks or meetings?	○ 11 = TIER<br>○ 12 = Population Council<br>○ 13 = Heartland Alliance<br>○ 14 = SHIPs for MARPs<br>○ 15 = Global Fund (SFH)<br>○ 16 = Other (If other, specify): ______________<br>○ 88 = Refusal<br>○ 99 = Don't know
11.08	In the last 12 months, have you participated in any talks or meetings related to HIV/AIDS with other MSM?	○ 01 = No information [End Questionnaire]<br>○ 02 = Health Facility<br>○ 03 = Peer Educator/workshop<br>○ 04 = School<br>○ 05 = Internet<br>○ 06 = Friends<br>○ 07 = Media<br>○ 88 = Refusal<br>○ 99 = Don't know
	11.09 Who hosted these talks or meetings with other MSM?	○ 01 = Heartland<br>○ 03 = CRH<br>○ 04 = Population council<br>○ 08 = FHI<br>○ 09 = SFH<br>○ 10 = APIN<br>○ 11 = TIER<br>○ 12 = Other (specify) ______________<br>○ 88 = Refusal<br>○ 99 = Don't know

End of questionnaire

"Thank you for taking the time to sit with me. I know we have gone through many questions and I really appreciate your time and openness. We are finished with the long part of the study. Do you feel ready to proceed with clinical care?"

Perform HCT (if Visit 1-7 and the participant was negative at previous visit or status unknown),
Or escort to Nurse Case Manager (if Visit 1-7 and the participant's HIV status is positive)
